# The use of eye-tracking technology as a tool to evaluate social cognition in people with an intellectual disability: a systematic review and meta-analysis

**DOI:** 10.1186/s11689-023-09506-9

**Published:** 2023-12-04

**Authors:** L. A. Jenner, E. K. Farran, A. Welham, C. Jones, J. Moss

**Affiliations:** 1 School of Psychology, University of Surrey, Surrey, UK; 2 School of Psychology, University of Birmingham, Birmingham, UK

**Keywords:** Eye-tracking, Social cognition, Intellectual disability, Genetic syndromes, Autism

## Abstract

**Background:**

Relatively little is known about social cognition in people with intellectual disability (ID), and how this may support understanding of co-occurring autism. A limitation of previous research is that traditional social-cognitive tasks place a demand on domain-general cognition and language abilities. These tasks are not suitable for people with ID and lack the sensitivity to detect subtle social-cognitive processes. In autism research, eye-tracking technology has offered an effective method of evaluating social cognition—indicating associations between visual social attention and autism characteristics. The present systematic review synthesised research which has used eye-tracking technology to study social cognition in ID. A meta-analysis was used to explore whether visual attention on socially salient regions (SSRs) of stimuli during these tasks correlated with degree of autism characteristics presented on clinical assessment tools.

**Method:**

Searches were conducted using four databases, research mailing lists, and citation tracking. Following in-depth screening and exclusion of studies with low methodological quality, 49 articles were included in the review. A correlational meta-analysis was run on Pearson’s *r* values obtained from twelve studies, reporting the relationship between visual attention on SSRs and autism characteristics.

**Results and conclusions:**

Eye-tracking technology was used to measure different social-cognitive abilities across a range of syndromic and non-syndromic ID groups. Restricted scan paths and eye-region avoidance appeared to impact people’s ability to make explicit inferences about mental states and social cues. Readiness to attend to social stimuli also varied depending on social content and degree of familiarity. A meta-analysis using a random effects model revealed a significant negative correlation (*r* = −.28, [95% CI −.47, −.08]) between visual attention on SSRs and autism characteristics across ID groups. Together, these findings highlight how eye-tracking can be used as an accessible tool to measure more subtle social-cognitive processes, which appear to reflect variability in observable behaviour. Further research is needed to be able to explore additional covariates (e.g. ID severity, ADHD, anxiety) which may be related to visual attention on SSRs, to different degrees within syndromic and non-syndromic ID groups, in order to determine the specificity of the association with autism characteristics.

Social cognition refers to the ability to spontaneously read and interpret social and emotional cues [1]. Social-cognitive abilities are conceptualised hierarchically, with visual social attention viewed as a necessary precursor for effective appraisal of mental states [2–4]. Eye-tracking technology has been used to detect early emerging differences in visual social attention in autistic people and their infant siblings. Examples include reduced gaze-following and inattention to social cues [5–9]. It is thought that these differences in visual social attention contribute to challenges with higher-level appraisal abilities (e.g. misinterpretation of facial expressions, mentalising difficulties) that are evident across the lifespan of some autistic people [10–12]. Social-cognitive differences have been shown to predict social difficulties in autistic children and adults without intellectual disability (ID) [13, 14]. Unfortunately, people with ID are often excluded from autism research despite high co-occurrence [15], and studies of social cognition are no exception. In this article, we begin by highlighting how eye-tracking technology could advance social-cognitive research for people with ID. We emphasise the importance of improved accessibility and sensitivity, with reference to the autism literature. A systematic review is then used to synthesise social-cognitive research which has used eye-tracking technology in ID. A meta-analysis was conducted to explore the relationship between visual social attention during these tasks and autism characteristics across ID groups.

## Social cognition and intellectual disability

Social functioning is inherent in the conceptualisation of ID, with evaluation of day-to-day social abilities being one of several core components used to determine a person’s global adaptive functioning, alongside IQ [16]. Autism^1^frequently co-occurs with ID (> 40% [16, 21]) and a number of genetic syndromes in which ID is central to the phenotype (e.g. fragile X, Cornelia de Lange, Prader-Willi syndrome), present with an increased prevalence of clinically significant autism characteristics [22–24]. However, relatively little is known about the development and profile of social-cognitive abilities among people with ID, particularly with regard to co-occurring autism.

Traditional measures of social cognition are typically demanding on language and domain-general cognitive abilities. The participant is shown a stimulus or vignette and asked to verbally identify a character’s thoughts, feelings and/or intentions. During these tasks, the participant is required to hold the stimuli and/or scenario in mind, understand a test question and provide a response. In autistic adults without ID, performance on such measures has been related to IQ [25]. It is therefore unsurprising that people with genetic syndromes associated with ID score relatively poorly when traditional social-cognitive measures are used [26]. Furthermore, performance on social-cognitive tasks in people with ID has been related to executive function (e.g. [26, 27]) and language (e.g. [28]) difficulties. Even in genetic syndromes (i.e. Williams syndrome) where social cognition has been thought to be a relative strength [29], social-cognitive strengths are primarily evident among those with a milder severity of ID [30, 31]. Together, this highlights the challenge of disentangling social-cognitive abilities from the language and domain-general cognitive difficulties which are central to ID when traditional measures are used.

Though social difficulties may be characteristic of ID [16], the nature of these difficulties and the degree to which they manifest in each genetic syndrome is highly variable. For instance, people with Down syndrome and Rubinstein-Taybi syndrome present with high levels of social motivation [32, 33], whereas Cornelia de Lange syndrome and fragile X syndrome are characterised by social anxiety and extreme shyness [34]. Notably, profiles of autism characteristics are highly heterogeneous and appear qualitatively different, often in very subtle ways, across genetic syndromes and when compared to non-syndromic^2^ autism [35]. This heterogeneity cannot be accounted for by degree of ID severity [22] and appears to reflect the broader behavioural phenotypes presented in specific genetic syndromes [36]. For instance, people with Down syndrome who score above threshold on autism screening tools are less withdrawn from their surroundings than those with non-syndromic autism—representing their high levels of social motivation [37]. Given the association between social cognition and social behaviour in autism [13, 14], it is possible that variable profiles of social-cognitive strengths and difficulties may also underly these heterogeneous profiles of autism characteristics in genetic syndromes associated with ID.

To further delineate autism profiles, Ellis and colleagues [38] measured the developmental sequence of early social-cognitive skills (i.e. intention reading) by using behavioural responses to basic goal-directed actions—suitable for children with ID and limited language. Relative to neurotypical children, children with Rubinstein-Taybi, Cornelia de Lange, and fragile X syndrome demonstrated similarly delayed acquisition of early social-cognitive skills as autistic children. However, children with these genetic syndromes did not pass tasks in the same order as autistic and neurotypical children. Performance was not related to general cognitive delay, pointing to an alternative mechanism which may be disrupting the sequence in which social-cognitive abilities are acquired. This study demonstrates that in genetic syndromes, behavioural phenotypes and related profiles of autism characteristics may be underpinned by divergent trajectories of social-cognitive development. However, conclusions are limited as behavioural observation lacks sensitivity to detect more subtle mechanisms underlying these social-cognitive processes within and across ID groups.

## Eye-tracking as a tool to evaluate social cognition in autism

In autism research, eye-tracking technology has become an increasingly popular method of studying early emerging differences in visual social attention [5, 7], which differentiate autistic and neurotypical people [6, 9]. Studies on autistic toddlers have found that reduced gaze towards people within social scenes [39], the eye region of faces [40] and increased preference for non-social (versus social) stimuli [41] is significantly correlated with greater severity scores on the Autism Diagnostic Observational Schedule (ADOS; [41]). These findings have also been evidenced among autistic children [42], adults [43] and in the broader autism phenotype [44, 45]. Significant correlations between visual social attention and autism characteristics have also been evidenced using screening questionnaires [46, 47], and changes in visual social attention have been associated with behavioural change over time [48].

A key benefit of eye-tracking technology is that paradigms can be devised which present participants with stimuli in a passive, free-viewing manner, without the need for explicit responses or verbal demands. Not only has this supported research on ‘markers’ of autism in infancy [8, 49], but has provided a more sensitive method of studying higher-level social-cognitive abilities. For example, anticipatory gaze has been used as a non-verbal measure of false-belief reasoning [50]. Similar to traditional false-belief measures (e.g. the Sally-Anne task; [51]), participants are shown a change-location scenario, where the location of an object is moved when the actor is not looking. Autistic adults are less likely to show anticipatory gaze towards where the actor last saw the object when they return, appearing to not anticipate the actor’s false-belief [50, 52, 53]. Interestingly, these adults were able to pass traditional false-belief measures which required a verbal response, suggesting their language ability and possibly other strategies (e.g. learning the ‘rules’) were able to compensate for underlying social-cognitive difficulties. These findings illustrate how eye-tracking can reduce the confound of language and domain-general cognition, even when measuring higher-level social-cognitive abilities—highlighting potential as an inclusive and accessible tool to evaluate social cognition in autistic people with few or no words [54, 55].

## The systematic review and meta-analysis

Eye-tracking technology is a sensitive and direct method of measuring social-cognitive abilities, independent of language and with reduced domain-general cognitive demands. Furthermore, there is evidence of an association between visual social attention and autism characteristics in autistic people and the broader autism phenotype. Despite extensive work in autism research, no review to our knowledge has explored how eye-tracking technology has been used to evaluate social cognition among people with ID. The aim of the systematic review was to provide an account of research which has used eye-tracking paradigms to study social-cognitive abilities in ID. A meta-analysis was used to explore whether visual social attention during these tasks correlated with degree of autism characteristics presented on clinical assessment tools. Synthesis of current research in this way is a necessary step to begin to evaluate the utility and feasibility of eye-tracking as a methodology to study social cognition and autism in ID.

## Methods

### Literature search

Following the Preferred Reporting Items for Systematic Reviews and Meta-analyses (PRISMA [56]) Statement, a systematic review was conducted. The key components for the search query were (1) intellectual disability and (2) eye-tracking. The intellectual disability component included terms for both syndromic (e.g. ‘genetic syndrome*’, ‘fragile X syndrome*’) and non-syndromic (e.g. ‘intellectual disab*’) groups. Where databases allowed, controlled vocabulary (e.g. Medical Subject Headings [MeSH]) was also included. Search terms were determined from an initial scoping of literature, followed by investigation of controlled vocabulary. Social cognition was not included as a separate component, as some eye-tracking terms describe social-cognitive abilities (e.g. ‘face scan*’). Peer review of the search strategy was conducted to improve the quality, using the PRESS guidelines [57]. The full systematic review search strategy and search queries were pre-registered and are available to access: https://osf.io/ktp2r/.

Searches were conducted in PsycINFO, MEDLINE, Embase and Web of Science. Filters for the databases were used where possible to include the following: (a) English language, (b) peer-reviewed and grey literature (c) published between 2000 and 2022 and (d) human participants. Only literature available in English was included to ensure consistency in definitions related to intellectual disability, eye-tracking and social cognition. Searches were also conducted through relevant ID research mailing lists, as well as forwards/backwards citation tracking.

### Inclusion and exclusion criteria

**Fig. 1:**
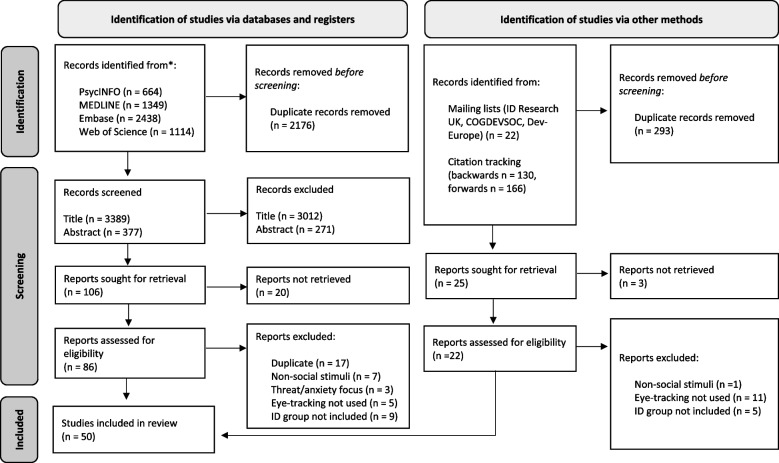
PRISMA (2020) flow diagram for systematic reviews

All identified records were pooled, and duplicates were removed (see Fig. 1). Titles and abstracts from identified records were screened using the following exclusion criteria: (1) studies that code eye gaze from observation or use a neuroimaging technique, rather than using eye-tracking technology, and (2) papers available only in a language other than English. To be included, papers needed to report empirical research. The title or abstract had to indicate that the method of data collection involved an eye-tracking paradigm which measured responses to social stimuli (e.g. emotional expressions, social scenes) or a social-cognitive task (e.g. false-belief reasoning). Studies which focused only on response to threat/anxiety (e.g. fearful faces) were not included, given the known interplay between anxiety and social functioning (e.g. in Williams syndrome [58]).

The dependent variable of interest was visual attention. Examples of variables include proportion of fixations towards areas of interest, overall dwell time and/or direction of first saccade. Studies included had at least one group of participants with syndromic or non-syndromic ID. Groups where associated ID and adaptive functioning is highly variable (e.g. autism, Klinefelter syndrome) were excluded if either clinical diagnosis of ID or an appropriate metric indicating ID (e.g. IQ < 70) was not reported. These ID-specific descriptors were not required for inclusion of groups where ID is core to the behavioural phenotype (e.g. fragile X syndrome). Participants could be of any age. Study design was not specified. Two independent reviewers screened the studies’ titles (*κ* = .84) and abstracts (*κ* = .87), indicating excellent reliability. In cases of disagreement, a third party was consulted.

#### Quality rating

A quality criteria checklist from Cross and Hare [59] was used, which was originally created based on reported best practice for behavioural phenotype methodology. Criteria have been adapted to ensure they are applicable to samples with non-syndromic ID (see Table 1).

**Table 1:** Quality appraisal checklist based on Cross and Hare (2013)

**Criteria**	**Quality rating**		
	0	1	2
1. Sample size?	Fewer than 15 participants	15+ participants	30+ participants
2. Recruitment?	Participants selected by clinicians or researcher	Participants recruited either through community outreach, a charity, school, or medical clinic	Multiple methods, multiple clinics, school, or multiple charities are used for recruitment
3. Diagnosis? ^a^	Diagnosis not confirmed	Diagnosis based on non-expert opinion or physical features	Confirmed clinical diagnosis or appropriate genetic/enzyme testing
4. Comparison group? ^b^	No comparison group	Comparison between non-genetically distinct group	Genetically or intellectually distinct comparison group
5. Methodology?	No validated or standardised measures are used	Use a validated and/or standardised assessment tool	Multiple standardised and/or validated measures are used alongside new measure, observations, or other methodology.
6. Appropriate statistics/ comparisons?	Data not analysed	Descriptive statistics are used	Appropriate comparative/correlative statistics are reported

^a, b^ Quality rating options adapted to be applicable for both non-syndromic and syndromic intellectual disability groups

For each of the criteria, the study was allocated a score of 0, 1 or 2 according to the degree to which the criterion was met. A score of 0 was also used when information was not stated or could not be assessed. The ‘developmental trajectory’ item included in the original Cross and Hare [59] checklist was removed due to it not being appropriate for the review aims, as is the case for other systematic reviews which have used the checklist [60, 61]. A total score of 0-12 can be achieved, with higher scores indicating greater quality. A quality rating in the upper tertial is recommended for study inclusion [59]. For the amended criteria used in this review, a rating in the upper tertial is indicated by a score of eight or more. One study was omitted [62] due to a methodological quality score below seven. Quality ratings were repeated for studies included in the meta-analysis (see Table 2), following the removal of criteria which were accounted for within the meta-analysis (i.e. sample size, appropriate statistics) or no longer relevant (i.e. comparison groups). In this instance, the maximum score was six.

**Table 2:** Overview of the studies included in the meta-analysis

**Authors**	**Quality**	**Group**	***N***	**Socially salient region of the stimuli**	**Measure of autism characteristics**
Benjamin et al. (2014) [63]	4	FXS	14	Proportion of gaze on target object pointed to by actor.	ADOS severity score
Cooke et al. (2019) [64]	2	PMS	15	Proportion of gaze on social (versus non-social stimuli).	ADOS severity score
Crawford et al. (2015a) [65]	6	FXS	12	Proportion of gaze on eye region of faces.	SCQ total score
Crawford et al. (2015b) [66]	6	CdLS	15	Proportion of gaze on eye region of faces.	SCQ total score
		RTS	16		
Crawford et al. (2016) [67]	6	FXS	15	Proportion of gaze on direct social (versus non-social) stimuli.	ADOS severity score
		CdLS	13	Proportion of gaze on direct social (versus non-social) stimuli.	SCQ total score
		RTS	18		
Crawford et al. (2017) [68]	5	FXS	11	Proportion of gaze on face of actors within a social scene.	ADOS severity score
Hall et al. (2015) [69]	4	FXS	51	Total duration of gaze on face of experimenter during social interaction.	SCQ total score
		nsID	19		
Hanley et al. (2013) [70]	6	WS	15	Proportion of gaze on eye region of faces.	SRS total score
Hong et al. (2017) [71]	5	AS	8	Proportion of gaze on social (versus non-social) videos.	SRS social motivation score
Hong et al. (2019) [71]	4	FXS	11	Proportion of gaze on social (versus non-social) videos.	SCQ total score
Klusek et al. (2019) [72]	6	FXS	24	Proportion of gaze on eye region of face.	ADOS severity score
Yi et al. (2015) [73]	5	nsID	26	Proportion of gaze on eye region of face.	GARS total score

The maximum quality rating score was six, following the removal of Cross and Hare (2013) criteria which were accounted for within the meta-analysis (i.e. sample size, appropriate statistics) or no longer relevant (i.e. comparison groups). Autism Diagnostic Observational Schedule (ADOS) [74]. Social Responsiveness Scale [SRS; [75]], Social Communication Questionnaire [SCQ; [76]], Gilliam Autism Rating Scale [GARS; [77]]

### Data extraction

Studies which met eligibility criteria were examined to extract data regarding the ID sample characteristics (i.e. ID aetiology, N, chronological age, general ability), exclusion criteria, comparison groups, social-cognitive domain measured, eye-tracking paradigm used and principal findings. Where possible, Pearson’s *r* value reporting the relationship between visual social attention and autism characteristics was also extracted (see Table 2). If a study measured autism characteristics, but this relationship was not explored, then a request was made to obtain Pearson’s *r* value from the authors via correspondence.

## Results

### Systematic review

The majority of paradigms measured expression discrimination (*N* = 16 [65, 66, 70, 78–90]; 31.37%) and social preference (*N* = 10 [64, 67, 71, 91–97]; 19.61%), whereas fewer investigated face recognition (*N* = 6 [73, 98–102]; 11.76%), social scene scanning (*N* = 8 [68, 80, 103–108]; 15.69%), gaze-following (*N* = 3 [63, 97, 109]; 5.88%), face scanning (*N* = 2 [69, 110]; 3.92%), attention to the eye region (*N* = 2 [72, 111]; 3.92%), overimitation (*N* = 2 [112, 113]; 3.92%), and false-belief reasoning (*N* = 1 [114]; 1.96%). Characteristics of the ID sample/s, comparison group/s, the eye-tracking paradigm and principal findings from each study are summarised in Table 3. Studies which used different eye-tracking paradigms to measure multiple social-cognitive domains are described separately.

**Table 3:** Overview of the reviewed studies which used eye-tracking to measure social cognition in intellectual disability

Author (date)			Quality rating	Participant characteristics					Eye-tracking paradigm	
				ID aetiology (N)	Chronological age (CA) Mean ± SD	Developmental level Mean ± SD		Comparison group(s) (N) ^*^matched	Methodology	Principle finding
**Expression discrimination.** Gaze towards salient facial features across different emotional facial expressions										
Campbell et al. (2010) [78]			10	22q11.2 deletion syndrome (17)	17.2 ± 3.2	FSIQ 72.8 ± 13.2		Neurotypical (17) *Gender, CA	35 images of human facial expressions (neutral, happy, sad, surprise, angry, fear, and disgust). Participants were asked to explicitly identify the expression viewed.	Across expressions, people with 22q11.2 deletion syndrome looked less at the eyes, more at the mouth, and had fewer fixations/shorter scan paths.
Crawford et al. (2015a) [65]			11	Fragile X syndrome (13)	19.7 ± 9.07	VABSr 357.9 ± 95.6		Autism (15) *VABSr	38 images of human facial expressions (happy, disgust) paired against neutral faces.	People with fragile X syndrome looked less at the eye region of neutral faces than autistic people. Both groups showed a spontaneous preference for emotional expressions.
Crawford et al. (2015b) [66]			11	Cornelia de Lange syndrome (15)	18.4 ± 9.8	VABSc 59.9 ± 25.0		* CA, gender, autism traits, VABSc	38 images of human facial expressions (happy, disgust) paired against neutral faces.	People with Cornelia de Lange and Rubinstein-Taybi syndrome looked similarly at the eye and mouth region and showed spontaneous preference for emotional expressions.
				Rubinstein-Taybi syndrome (17)	17.3 ± 10.1	VABSc 58.5 ± 15.1				
Dalton et al. (2008) [79]			8	Fragile X syndrome (9)	20.7 ± 2.8	FSIQ 66.1 ± 23.8		Autism (14)	Images of human emotional (happy, fear, disgust) and neutral facial expressions. Participants were asked to explicitly identify the expression viewed.	People with fragile X syndrome looked similarly at facial features as autistic people, but less at the eye region than neurotypical people.
								Neurotypical (15)		
Debladis et al. (2019) [80]			12	Prader-Willi syndrome (39)	28.0 ± 8.0	FSIQ 57.0 ± 10.0		Neurotypical (20)	35 images of human emotional (happy, sad, fear) and neutral facial expressions. Participants were asked to explicitly identify the expression viewed.	People with PWS (mUPD) looked less at the eye region and had more difficulty with spontaneous discrimination of emotional expressions than those with PWS (deletion) and neurotypical controls.
Djukic et al. (2014) [81]			11	Rett syndrome (37)	10.0 ± 7.7	VABSc 45.9 ± 12.5		Neurotypical (34) *Gender, CA	12 images of human emotional (happy, sad, fear) and neutral facial expressions. Participants were asked to explicitly identify the expression viewed.	People with Rett syndrome had difficulty spontaneously discriminating emotional expressions and spent less time fixating on salient facial features, unlike the neurotypical group.
Farzin et al. (2009) [82]			7	Fragile X syndrome (16)	17.0 ± 6.8	FSIQ 58.4 ± 9.8		Neurotypical (16) *Gender, CA	60 images of human facial expressions (calm, happy, fear) and 60 scrambled versions of the faces. Participants were asked to explicitly identify the expression viewed.	People with fragile X syndrome showed increased pupil reactivity to facial expressions and looked less at the eye region across faces, compared to neurotypical comparison groups.
Farzin et al. (2011) [83]			10	Fragile X syndrome (15)	18.8 ± 10.7	FSIQ 57.5 ± 14.5		Neurotypical (20) *CA	Replication of Farzin et al (2009).	High test-retest reliability of gaze and pupillometry measures.
Franchini et al. (2016) [84]			11	22q11.2 deletion syndrome (35)	18.2 ± 5.9	FSIQ 57.0 ± 10.0		Neurotypical (20) *Gender, CA	48 videos of dynamic avatar facial expressions (anger, fear, happy, sad). Participants were asked to explicitly identify the expression viewed.	People with 22q11DS were slower to recognise emotional expressions than neurotypical controls. They also spent less time looking at the nose during happy and fearful faces.
Gomez et al. (2020) [85]			11	Williams syndrome (22)	12.4 ± 3.8	NA		Neurotypical (21) *Gender, CA	48 pairs of images of avatars with ‘trustworthy’ and ‘untrustworthy’ expressions. Spontaneous preference was measured alongside explicit appraisal of trustworthiness.	People with Williams syndrome did not exhibit a preference for trustworthy faces, unlike neurotypical people.
Hanley et al. (2013) [70]			11	Williams syndrome (15)	21.9 ± 9.3	FSIQ 72.8 ± 13.2		Neurotypical (15) *CA	Image and video depictions of eight mental states (e.g. relieved, surprised, worried) expressed by an actor. Participants were asked to explicitly identify the actor’s mental state.	In contrast to both comparison groups. people with Williams syndrome looked less at salient facial features whilst making judgements about mental states.
								Neurotypical (14) *BPVS		
Kirk et al. (2013) [86]			10	Williams syndrome (13)	23.6 ± 6.9	BPVSr 132.0 ± 18.9		Neurotypical (13) *CA	20 images of human emotional (angry, happy, sad, fear) and neutral facial expressions. Participants were asked to explicitly identify the expression viewed.	People with Williams syndrome fixated on the eye region of faces similarly to comparison groups. High levels of behavioural anxiety associated with reduced gaze towards the eye regions of threatening facial expressions in Williams syndrome.
								Neurotypical (13) *BPVS		
McCabe et al. (2011) [87]			10	22q11.2 deletion syndrome (18)	17.4 ± 3.1	FSIQ 73.8 ± 13.6		Neurotypical (17) *Gender, CA	28 images of human emotional (happy, sad, surprise, disgust, afraid, angry) and neutral faces. Discrimination of 35 non-face stimuli (weather scenes) also measured. Participants were asked to explicitly identify the expression/weather viewed.	People with 22q11.2 deletion syndrome demonstrate fewer and longer fixations across facial expression and non-face (weather scenes) stimuli compared to a neurotypical comparison group.
McCabe et al. (2013) [88]			7	22q11.2 deletion syndrome (20)	16.8 ± 3.7	FSIQ 72.1 ± 13.0		Autism (14) *CA	28 images of human emotional (happy, sad, surprise, disgust, afraid, angry) and neutral faces. Discrimination of 35 non-face stimuli (weather scenes) also measured. Participants were asked to explicitly identify the expression/weather viewed.	For faces, the 22q11.2 deletion syndrome and autism groups demonstrated lower emotion recognition accuracy and fewer fixations compared to the neurotypical group. People with 22q11.2 deletion syndrome looked more at weather scenes than autistic people yet had more difficulty with explicit appraisal than autistic and neurotypical groups.
								Neurotypical (20) *CA		
Porter et al. (2010) [89]			9	Williams syndrome (16)	25.1 ± 11.7	FSIQ 61.0 ± 15.0		Neurotypical (16) *Gender, FSIQ	24 images of human emotional (happy, sad, surprise, disgust, afraid, angry) and neutral faces. Participants were asked to explicitly identify the expression viewed.	People with Williams syndrome did not look at the eye region faster than neurotypical people. But, once attended people with Williams syndrome spent more time looking at the eye region, People with William syndrome’s scan paths were similar across the different facial expressions but showed most difficulty with explicit recognition of anger.
Shaw & Porter (2013) [90]			11	Fragile X syndrome (16)	24.8 ± 12.9	FSIQ 64.0 ± 13.7		Neurotypical (16) *CA, gender	24 images of human emotional (happy, sad, surprise, disgust, afraid, angry) and neutral faces. Participants were asked to explicitly identify the expression viewed.	Whilst people with FXS displayed reduced fixations on the eyes and scanned facial expressions significantly differently compared to the CA-matched neurotypical comparison group, scan paths were similar to the MA-matched neurotypical comparison group.
								Neurotypical (16) *MA, gender		
**Facial recognition.** Gaze towards novel faces when presented alongside a familiar face.										
Glaser et al. (2010) [98]			12	22q11.2 deletion syndrome (26)	12.4 ± 1.9	FSIQ 74.2 ± 11.8		Neurotypical (22) *CA	Paired images of human faces with neutral expressions, with either configural differences (30 trials) or featural differences (30 trials). Participants were asked to identify if the faces were the ‘same’ or ‘different’ to each other.	Few differences in explicit recognition accuracy between people with 22q11.2 deletion syndrome and neurotypical comparison group. People with 22q11.2 deletion syndrome spent more time looking at the mouth region than the eye region than those with non-syndromic ID.
				Non-syndromic ID (17)	11.5 ± 2.7	FSIQ 68.4 ± 10.2				
Guillory et al. (2021) [99]			9	Phelan-McDermid syndrome (8)	9.2 ± 3.4	DQ 35.8 ± 19.8		Neurotypical (26) *CA	Identical images of human faces (happy) presented in pairs (familiarisation), which switched with an image of a novel face (pre-switch). The two images then swap sides (post-switch). Nine trials in total. Repeated with non-social images.	Rate of looking back-and-forth between images was lowest in the non-syndromic ID (+ autism) group. Across indices, people with Phelan-McDermid syndrome (+ autism) looked more similarly to people with Phelan-McDermid syndrome-alone, rather than the non-syndromic ID (+ autism) group.
				Phelan-McDermid syndrome (+ autism) (14)	9.9 ± 4.2	DQ 17.3 ± 12.2				
				Non-syndromic ID (+ autism) (7)	7.8 ± 2.7	DQ 45.5 ± 21.1				
Rose et al. (2013) [100]			10	Rett syndrome (27)	10.6 ± 6.8	VABSc 44.2 ± 10.1		Neurotypical (30) *Gender, CA	Identical images of human faces (happy) presented in pairs (familiarisation), which switched with an image of a novel face (pre-switch). The two images then swap sides (post-switch). Nine trials in total. Repeated with non-social images.	Recognition of novel faces was poorer in Rett syndrome compared to neurotypical comparison group. People with Rett syndrome’s gaze was characterised by fewer and longer fixations. They all tended to ignore the mouth/nose region.
Yi et al. (2014) [101]			11	Non-syndromic ID (22)	23.6 ± 3.1	CRTr 23.8 ± 8.6		Neurotypical (28) *CA	74 images of neutral human faces: 37 own race and 37 other race. Presented individually, followed by same/different face. Participants were asked to identify if the faces were the ‘same’ or ‘different’ to each other.	Non-syndromic ID (+ autism) group looked more at the nose region. Non-syndromic ID groups scanned the whole face less than neurotypical comparison group.
				Non-syndromic ID (+ autism) (19)	20.8 ± 3.3	CRTr 23.7 ± 9.4				
Yi et al. (2015) [73]			11	Non-syndromic ID (26)	23.0 ± 3.1	CRTr 22.0 ± 8.8		Neurotypical (30) *CA	36 images of neutral human faces. Presented individually, followed by same/different face. Participants were asked to identify if the faces were the ‘same’ or ‘different’ to each other.	Non-syndromic ID groups displayed better recognition of own race faces. All groups displayed more looking at the eye region of other-race faces relative to own race faces.
				Non-syndromic ID (+ autism) (24)	20.7 ± 3.9	CRTr 22.4 ± 9.0				
Zaharia et al. (2018) [102]			12	22q11.2 deletion syndrome (time 1) (75)	12.8 ± 3.6	FSIQ 72.0 ± 11.3		Neurotypical (time 1) (84) *CA	Paired images of human faces with neutral expressions, with either configural differences (30 trials) or featural differences (30 trials). Participants were asked to identify if the faces were the ‘same’ or ‘different’ to each other.	People with 22q11.2 deletion syndrome look more at the mouth region and demonstrate restricted scan paths, with a reduced number of transitions between faces and longer fixations compared to the neurotypical comparison group. Similar scan paths evident across time 1 and 2 in 22q11.2 deletion syndrome.
				22q11.2 deletion syndrome (time 2) (36)	16.5 ± 3.1	FSIQ 69.1 ± 11.6		Neurotypical (time 2) (30)*CA		
**Social preference.** Proportion of gaze towards paired social versus non-social (e.g. geometric shapes) stimuli.										
Cooke et al. (2019) [64]		8		Phelan-McDermid syndrome (15)	8.9 ± 0.8	NA	Autism (24)		8 trials in which a human face was displayed among an array of four non-social (e.g. car, bird) images.	The majority of children with Phelan-McDermid syndrome and autistic children showed reduced: 1) first looks to the face and 2) less overall looking at face, compared to neurotypical children.
							Neurotypical (27)			
Crawford et al. (2016) [67]		11		Fragile X syndrome (15)	18.2 ± 5.6	VABSc 51.3 ± 17.4	Autistic and neurotypical children included in separate study to establish baseline.		Paired videos of actor (social) or object (non-social) moving towards/away from the camera. 28 trials in total.	Autistic people looked less at social versus non-social videos only when stimuli were moving towards them. Individuals in the three genetic syndrome groups showed similar looking-time but differences in fixation latency for social stimuli moving towards them.
				Cornelia de Lange syndrome (14)	20.9 ± 11.9	VABSc 47.9 ± 16.0				
				Rubinstein-Taybi syndrome (19)	24.2 ± 8.6	VABSc 47.8 ± 14.6				
Hirai et al. (2016a) [92]		10		Williams syndrome (21)	16.2 ± 7.1	RCPMr 18.3 ± 5.0	Neurotypical (21) *RCPMr		120 trials in which non-social images (e.g. clock, apple) presented in array alongside: 1) a human face, 2) a butterfly, 3) a face and butterfly, 4) neither face nor butterfly. Participants asked to explicitly identify when butterfly was present.	Unlike neurotypical comparison groups, people with Williams syndrome looked more at the face during later search stages. Slower explicit recognition of the butterfly correlated with increased fixation on face in Williams syndrome.
							Neurotypical (21) *CA			
Hirai et al. (2016b) [93]		9		Williams syndrome (17)	16.7 ± 6.5	RCPMr 18.4 ± 5.1	Neurotypical (17) *RCPMr		120 trials in which non-social images (e.g. clock, apple) presented in array alongside: 1) an inverted human face, 2) a butterfly, 3) an inverted face and butterfly, 4) neither face nor butterfly. Participants asked to explicitly identify when butterfly was present.	When the butterfly and inverted face were present in the same search array, explicit recognition of the butterfly was similar in Williams syndrome relative to neurotypical comparison groups. Neither group looked preferentially towards inverted faces.
							Neurotypical (17) *CA			
Hong et al. (2017) [93]		9		Angelman syndrome (8)	12.4 ± 10.7	VABSc 44.8 ± 20.7	Neurotypical (N not reported) *Gender, CA		Three trials in which a video clip of children interacting (social) was paired with geometric shapes (non-social).	Relative to neurotypical comparison groups, Angelman syndrome looked less at social stimuli, and showed increased pupil dilation for non-social stimuli. No difference between people with Angelman syndrome and autistic children.
							Autism (N not reported) *Gender, CA			
Hong et al. (2019) [71]		10		Fragile X syndrome (17)	16.6 ± 6.1	VABSc 61.2 ± 12.0	Neurotypical (17) *Gender, CA		Three trials where a video clip of children interacting (social) was paired with geometric shapes (non-social). Also, twelve trials in which an image of face was paired with scrambled (/geometric) face.	Whilst autistic comparison groups showed significantly less social preference, people with fragile X syndrome displayed social preference similar to the neurotypical comparison group.
							Autism (17) *Gender, CA			
Ponson et al. (2018) [94]		7		Phelan-McDermid syndrome (18)	12.7 ± 9.2	DQ 25.4 (10-55)	Neurotypical (N not reported)		Ten trials, in which an image of a neutral face (social) was paired with an object (non-social).	Unlike the neurotypical comparison group, people with Phelan-McDermid syndrome showed increased pupil dilatation when viewing the non-social versus social stimuli.
							Autism (N not reported)			
Riby & Hancock (2009a) [95]		9		Williams syndrome (14)	8.75 - 28.0	RCPMr 14	Neurotypical (14) *RCPMr		Images include 1) scrambled pictures containing faces (20 trials) and 2) pictures of scenes with embedded faces (9 trials) or no faces (9 trials).	People with Williams syndrome showed prolonged face gaze across tasks relative to neurotypical comparison group, whereas autistic comparison group showed reduced face gaze.
							Autism (14) *RCPMr			
Schwartzman et al. (2015) [96]		7		Rett syndrome (14)	12.4 ± 7.3	NA	Neurotypical 17)		Three images: 1) two children, 2) human face (happy) and 3) child and clock.	Proportion of fixations on social stimuli was higher in Rett syndrome relative to comparison groups.
							Autism (11)			
Vivanti et al. (2017a) [97]		7		Williams syndrome (21)	4.3 ± 1.4	VABSc 69.9 ± 9.8	Neurotypical (20) *CA		Five images of scene including social and non-social target. Three videos of paired social and non-social stimuli moving in unison.	Williams syndrome and neurotypical comparison group looked more at social stimuli compared to autistic group.
						DQ 56.1 ± 16.5				
							Autism (36) *CA, DQ			
**Social scene scanning.** Gaze allocation (e.g. people, objects, background) within a social scene.										
Crawford et al. (2017) [68]	8			Fragile X syndrome (11)	26.3 ± 9.1	BPVSr 87.0 ± 27.2	Neurotypical (11) *BPVSr		Twenty images of scenes where human actors engaged in natural activities.	Looking patterns were similar in fragile X syndrome and neurotypical comparison group. However, in fragile X syndrome gaze upon the face was associated with heightened anxiety and fewer social communication difficulties.
Debladis et al. (2019) [80]	12			Prader-Willi syndrome (39)	28.0 ± 8.0	FSIQ 57.0 ± 10.0	Neurotypical (20)		Three video clips of actors interacting whilst a third is standing in the background.	People with Prader-Willi syndrome looked similarly at social scenes as neurotypical people, but their gaze patterns became more atypical as social content increased.
Guy et al. (2020) [103]	11			Fragile X syndrome (6)	14.3 ± 2.0	BPVSr 126.3 ± 23.5	Neurotypical (15) *CA		Social scenes including a social (32 trials) and non-social (32 trials) distractor. Scenes included a unique target. Participants were asked to explicitly report when they found the target.	People with fragile X syndrome increased first looks to the target in later trials. However, overall attention to nonsocial distractor was higher in fragile X syndrome relative to comparison groups.
							Neurotypical (16) *BPVSr			
						RCPMr 22.3 ± 4.5				
							Neurotypical (16) *RCPMr			
Liang & Wilkinson (2017) [104]	9			Down syndrome (10)	19.0 ± 6.0	PPVTr 51	Autism (10) *CA, PPVTr		Sixteen pairs of images of social scenes, with two (8 trials) or three (8 trials) people. Each pair depicted an interaction where sharing was present and absent.	Gaze patterns were similar across groups. People with Down syndrome were quicker than comparison groups to view image where sharing was present.
							Neurotypical (10) *CA			
Riby & Hancock (2008) [105]	10			Williams syndrome (16)	17.5 ± 6.3	RCPMr 13.0 ± 4.0	Neurotypical (10) *CA		Twenty images of scenes where human actors engaged in natural activities.	Whilst autistic people spend less time viewing people/faces than is typical, people with Williams syndrome demonstrate exaggerated fixations towards the eyes.
							Neurotypical (16) *RCPMr			
							Autism (20)			
Riby & Hancock (2009b) [106]	9			Williams syndrome (16)	17.5 ± 6.3	RCPMr 16.0 ± 5.0	Neurotypical (10) *CA		Twenty images from actors interacting. The position and number of characters varied. Video clips including human actors (3 trials) and cartoons (3 trials).	Autistic people attended to faces less than was typical, whereas individuals with Williams syndrome attended to the face for longer than is typical. Atypical gaze behaviours in Williams syndrome were restricted to human actors.
							Neurotypical (16) *RCPMr			
							Autism (20)			
Wilkinson & Light (2014) [107]	7			Down syndrome (5)	17.8 ± 2.1	PPVTr 27	Neurotypical (5)		Eight images of an actor in a scene.	Fixation towards actor was similar across all groups.
				Non-syndromic ID (3)	15.5 ± 0.7	PPVTr 36				
							Autism (5)			
Williams et al. (2013) [108]	11			Williams syndrome (14)	22.2 ± 8.7	FSIQ 56.0 ± 13.1	Neurotypical (14) *Gender, CA		Eighteen images of social scenes including an actor who was presented centrally or peripherally.	People with fragile X syndrome avoided the actor presented centrally, at least initially. People with Williams syndrome took longer than is typical to disengage from actor.
				Fragile X syndrome (14)	23.0 ± 10.5	FSIQ 64 ± 14.7				
							Neurotypical (14) *Gender, FSIQ			
**Gaze-following.** Gaze towards a target object following an actor’s gaze cue.										
Benjamin et al. (2014) [63]	8			Fragile X syndrome (11)	7.7 ± 1.9	PPVTr 70.71 ± 34.5	Autism (17) *PPVTr		Video of an actor presenting one of two novel objects. The actor gazes towards the target object and then points towards the target object. Two novel objects presented within each of the four trials.	The fragile X group showed significantly increased face gaze rather than gaze-following towards the target object, whereas the comparison groups showed similar amounts of relative gaze towards the face and objects. The act of pointing increased gaze toward the target objects compared to when there was no pointing in all groups.
						Leiter NVIQ 58.6 ± 13.5	Neurotypical (18) *PPVTr			
Riby et al. (2013) [109]	10			Williams syndrome (14)	13.5 ± 5.8	RCPMr 15 ± 5	Neurotypical (14) *RCPMr		Fourteen different images of an actor in a complex scene (e.g. office). The actor’s gaze was directed to a target item in the scene. Target object was presented alongside a plausible and implausible target. Participants were asked to explicitly identify the target object.	People with Williams syndrome looked more at the face and eye region compared to comparison groups. When cued to follow gaze, people with Williams syndrome looked towards the target, unlike autistic people who had greater difficulty identifying the target object.
							Autism (24) *RCPMr			
Vivanti et al. (2017a) [69]	11			Williams syndrome (21)	4.3 ± 1.4	VABSc 69.9 ± 9.8	Neurotypical (20) *CA		Six videos of an actor gazing towards one of two objects. The actor looks up to establish direct gaze and then turns head to gaze at the target object.	People with Williams syndrome looked less at the target object compared to neurotypical comparison group.
							Autism (35) *CA, DQ			
						DQ 56.1 ± 16.5				
**Attention to the eye region.** Gaze specifically towards or away from the eye region of neutral faces.										
Kleberg et al. (2022) [111]	8			Williams syndrome (37)	23.4 ± 12.2	NA	Neurotypical infants (37)		Image of human face displaying an angry, happy, or neutral facial expression. 60 trials in total, 30 each condition. Fixation cross, so point of gaze towards eyes, then mouth (eye cued condition and mouth cued condition).	People with Williams syndrome were less likely, and slower, to orient to the eyes compared with neurotypical comparison groups of all ages except infants.
							Neurotypical children (44)			
							Neurotypical adolescents (36)			
							Neurotypical adults (50)			
Klusek et al. (2019) [72]	11			Fragile X syndrome (24)	19.3 ± 2.7	FSIQ 39.4 ± 5.8	Neurotypical (23) *CA		Video of avatar displaying gaze which is directed (16 trials) or averted (16 trials).	People with fragile X syndrome had shorter first fixation on the eyes relative to the neurotypical comparison group. Gaze direction (directed/averted) did not change orienting to the eye region in either group.
**Face scanning.** Gaze towards salient facial features whilst speaking.										
D’Souza et al. (2015) [115]	8			Down syndrome (infant) (22)	1.4 ± 0.2	MSELma 8.5 ± 2.5	Neurotypical (25) *MSELma		Paired video clips of human actor talking. The mouth movement was either congruent or incongruent to auditory stimuli heard.	Toddlers with fragile X and Williams syndrome who had a relatively large receptive vocabulary made more fixations to the eyes (rather than the mouth) of the incongruent face. In Down syndrome, fixations to the actor’s overall face predicted vocabulary size.
				Down syndrome (toddler) (21)	2.4 ± 0.6	MSELma 15.9 ± 4.5				
				Williams syndrome (infant) (12)	1.3 ± 0.2	MSELma 8.7 ± 1.9				
				Williams syndrome (toddler) (25)	2.5 ± 0.7	MSELma 16.1 ± 4.5				
				Fragile X syndrome (toddler) (14)	2.9 ± 0.7	MSELma 15.3 ± 4.4				
Hall et al. (2015) [69]	10			Fragile X syndrome (51)	20.2 ± 3.8	VABSc 58.5 ± 23.5	CA, VABSc, SCQ		An experimenter sat opposite the participant engaging with the participant in conversation about familiar topics (e.g. friends, family, hobbies). Participant was prompted to maintain eye contact with experimenter.	Participants with fragile X syndrome spent significantly less time looking at the face, including eyes, nose, and mouth individually, and had shorter episodes (and longer inter-episodes) of social gaze than comparison group.
				Non-syndromic ID (19)	19.4 ± 2.9	VABSc 57.7 ± 16.8				
**Overimitation.** Gaze allocation during video demonstration of an action by an actor.										
Vivanti et al. (2016) [112]	7			Williams syndrome (21)	4.4 ± 1.4	VABSc 69.9 ± 10.1	Autism (36) *VABSc, DQ		Eight videos of actor performing an action with one of eight objects presented on a table. Two conditions: 1) playful (4 trials) and 2) neutral (4 trials).	Unlike autistic children, those with Williams syndrome increased their gaze towards actor who was demonstrating the action in a socially engaging manner.
						DQ 56.44 ± 16.9				
Vivanti et al. (2017b) [113]	7			Williams syndrome (18)	4.4 ± 1.4	VABSc 69.9 ± 9.8	Autism (36) *CA, DQ		Three videos in which an actor demonstrated a causally relevant and irrelevant action.	Children with Williams syndrome and neurotypical children were more likely to increase their attention to the actor’s face during demonstration of causally irrelevant actions, compared to autistic children.
						DQ 56.1 ± 16.5				
**False-belief reasoning.** Anticipatory gaze towards the location an actor saw an object last.										
Van Herwegen et al. (2015) [114]	9			Williams syndrome (14)	7.5 ± 1.6	RCPMr 13.3 ± 2.4	Neurotypical (15) *CA Autism (13) *CA		Change location false-belief reasoning task, where an object is moved whilst the actor leaves the room. Participants were asked to explicitly identify where the actor would look when they returned.	Unlike neurotypical who looked longer at the original location of the object upon the actors return, children with Williams syndrome and autistic children do not show such contrasts. Children with Williams syndrome spend longer looking at the actor.
						BPVSr 49.3 ± 18.4				

*BPVSr* British Picture Vocabulary scales raw score, *CRTr* Combined Raven Test raw score, *DQ* Developmental Quotient, *FSIQ* Full-scale Intelligence Quotient, *MSELma* Mullen Scales of Early Learning Mental Age, *NVIQ* Leiter Non-verbal Intelligence Quotient, *PPVT* Peabody Picture Vocabulary Test raw score, *VABSr* Vineland Adaptive Behaviour scales raw score, *VABSc* Vineland Adaptive Behaviour scales composite score, *RCPMr* Ravens Coloured Progressive Matrices raw score. Coloured circles indicate studies which used the same methodology

Data from these 49 studies were qualitatively synthesised to provide an account of (1) the ID sample characteristics and exclusion criteria, and (2) atypical visual social attention as an indicator of social-cognitive differences. These are presented in narrative form, to provide discussion regarding the inclusivity, accessibility, and sensitivity of eye-tracking technology as a measure of social cognition in ID.

#### Sample characteristics and exclusion criteria

Samples included those with Williams syndrome (*N* = 17; 29.31%), fragile X syndrome (*N* = 14; 24.14%), 22q11.2 deletion syndrome (*N* = 6; 10.34%), non-syndromic ID (*N* = 6; 10.34%), Rett syndrome (*N* = 3; 5.17%), Down syndrome (*N* = 3; 5.17%), Phelan-McDermid syndrome (*N* = 3; 5.17%), Cornelia de Lange syndrome (*N* = 2; 3.45%), Rubinstein-Taybi syndrome (*N* = 2; 3.45%), Angelman syndrome (*N* = 1; 1.72%) and Prader-Willi syndrome (*N* = 1; 1.72%). People with Williams syndrome were included in studies evaluating several social-cognitive domains, whilst the focus of social-cognitive research was much narrower for other populations. Sample size varied across studies, ranging from 3 to 75 participants (M = 20, SD = 11.25), reflecting the rarity of the genetic syndromes studied. Thus, a common caveat of the data presented going forward is small sample sizes (see Table 3). To attain a larger sample, most studies included a wide age range of both children and adults. Few studies focused on children under six years old [97, 112, 113], or toddlers and infants [110] specifically.

Studies in which full-scale IQ and adaptive functioning were measured reported samples characterised predominantly by those with a mild-moderate degree of ID (see Table 3)*.* The mean full-scale IQ reported for ID samples ranged from 39.4 (± 5.82) to 73.8 (± 13.6), and adaptive behaviour composite scores ranged from 44.2 (± 10.1) to 69.9 (± 10.1). Hong and colleagues [93] focused on participants with Angelman syndrome, a genetic syndrome characterised by severe to profound ID, and reported that over half of their sample (*N* = 9) were unable to complete the eye-tracking task. In this study, adaptive functioning did not distinguish participants who engaged in the eye-tracking task from those who did not. Rather, unsuccessful eye-tracking was significantly associated with higher levels of hyperactivity and higher scores on the social motivation subscale of the SRS, indicating greater social motivation difficulties. The authors suggest measurement of these traits could be used as screening criteria to determine participant eligibility.

Challenges obtaining sufficient calibration (5- or 9-point) were commonly reported, leading to the exclusion of participants in both ID and comparison groups [68, 93, 95, 100, 105, 106, 109]. Inadequate number of fixations (e.g. on more than 40% of trials [68]) due to difficulties sustaining attention also led to the exclusion of a small number of participants [68, 70, 71, 79, 80, 95, 100, 105]. In addition, visual impairments (e.g. strabismus) [65, 70, 79, 86, 95, 109] and physical disability (e.g. scoliosis; [100]) were common reasons for exclusion. None of the studies provided metrics to describe the quality of the eye movement data obtained from the included (or excluded) participants.

#### Atypical visual social attention as an indicator of social-cognitive differences

Compared to neurotypical groups with similar chronological age and/or developmental level^3^, people with ID often had more difficulty spontaneously discriminating different emotional expressions (e.g. fragile X syndrome [65], Cornelia de Lange and Rubinstein-Taybi syndromes [66], Williams syndrome [85, 89]) and recognising novel faces (e.g. Rett syndrome, [100], 22q11.2 deletion syndrome [102]). People with ID also had more difficulty with following gaze (e.g. Williams syndrome [109]), fragile X syndrome [63]) and implicit anticipation of other people’s beliefs and mental states (e.g. Williams syndrome [70, 114]) than neurotypical children with similar chronological age and/or developmental level. The visual attention data which indicate these social-cognitive differences are discussed in further detail below, according to three key themes which were prominent within the reviewed literature: (a) limited exploration of social stimuli, (b) eye region avoidance and (c) response to familiarity and social content.

- Limited exploration of social stimuli. Exaggerated fixations towards the eyes and face were reported in Down syndrome [104, 107, 110] and Williams syndrome [95, 97, 105, 106, 108, 112, 114] with an opposite looking pattern described in autistic comparison groups and those with fragile X syndrome with similar chronological age and/or developmental level. However, people with Down syndrome [110] and Williams syndrome [86] spent less time fixating on salient facial features when compared to neurotypical comparison groups with similar chronological age and/or developmental level; even when prompted to identify the expression viewed (in Williams syndrome [70]).

In Williams syndrome, reduced gaze towards facial features has been attributed to longer time taken to first fixate on the face [92, 108] and eyes [89, 111]. Once attended, people with Williams syndrome were less likely to disengage from these regions than neurotypical comparison groups with similar chronological age and/or developmental level. These ‘sticky fixations’ [114] had implications for recognition and interpretation of social cues. For example, children with Williams syndrome performed similarly to chronological age matched autistic children on an implicit false-belief reasoning task, as they remained fixated on the actor, rather than anticipating the object would be retrieved from where the actor saw it last, as was demonstrated in neurotypical children [114]. Children with Williams syndrome also had difficulty gaze-following, as they did not disengage their fixation from the face to follow the cued object, only doing so once prompted verbally [109]. When shown trustworthy and untrustworthy faces side-by-side, people with Williams syndrome spent longer fixating on one face in the pair, and reduced transitions between faces—showing no preference for either face type (unlike neurotypical groups matched on chronological age who prefer trustworthy faces [85]).

When compared to neurotypical groups matched on chronological age, people with 22q11.2 deletion syndrome also demonstrated shorter scan paths and fewer fixations to salient features of the face [78, 84, 88]. However, restricted scan paths were not face-specific in 22q11.2 deletion syndrome [87]. During facial recognition tasks, people with 22q11.2 deletion syndrome look longer at one face in the pair, and evidence reduced transitions between faces than chronological age matched neurotypical groups [102]; however, this was also evident for pairs of nonsocial stimuli [87]. Similar findings were also described in Rett syndrome [81, 100].

(b) Eye-region avoidance. In fragile X syndrome, people demonstrated shorter initial [72] and overall [71, 82, 83] fixations to the eye region of faces when compared to chronological age matched neurotypical groups, appearing similar to autistic people [65, 79] and those with non-syndromic ID [73, 101]. Even when prompted to maintain eye contact, people with fragile X syndrome more frequently avoided fixating on the eye region than those with non-syndromic ID [69]. Interestingly, people with fragile X syndrome showed reduced fixations to the eye region across conditions in which gaze direction (averted/directed) was manipulated [72]. This persistent avoidance of the eye region may be why children with fragile X syndrome remained fixated on the face during gaze-following trials (unlike autistic and neurotypical children matched on verbal ability, who followed gaze towards the target object). Instead, pointing increased saccades towards a target object in fragile X syndrome [63].

In a number of studies, reduced looking at the eye region of faces was related to less accurate emotional discrimination and/or facial recognition. These findings were evident in Williams syndrome [70], fragile X syndrome [90], 22q11.2 deletion syndrome [98] and non-syndromic ID [73, 101]. An exception was identified in people with Prader-Willi syndrome, where people with the maternal uniparental disomy variant demonstrated overall reduced proportions of fixations to the eye region compared to those with paternal deletion variant, yet both groups showed similarly poor recognition accuracy for faces and emotional expressions [80].

(c) Familiarity and social content. Syndrome-specific differences in perceptual capture and engagement whilst viewing social scenes appeared to be driven by degree of familiarity and the nature of the social content depicted. For instance, proportion of fixations across trials on actors in social scenes was similar in fragile X syndrome and neurotypical children comparable on receptive language [68]. However, when earlier and later trials were compared, those with fragile X syndrome were initially hesitant to fixate on an actor within a social scene [103]. Likewise, those with fragile X syndrome fixated less on an actor presented centrally in a scene, at least initially; this difference was not evident when the actor in the stimuli was located peripherally (in contrast to Williams syndrome;[108]). When viewing dynamic stimuli, the direction in which an actor was moving (towards/past) did not change the latency of fixation or overall dwell time in either fragile X syndrome or Rubinstein-Taybi syndrome, unlike autistic children and those with Cornelia de Lange syndrome, who were slower to fixate, and fixated less, on the actor moving towards them [67].

In Prader-Willi syndrome, exploration of social scenes became more atypical as the social content increased [80]. In contrast, children with Down syndrome were quicker to fixate on actors within a social scene than those with non-syndromic ID and autistic children [107], particularly when there were three actors depicted (compared to two) and sharing was occurring in the scene [104]. Similarly, people with Williams syndrome looked longer at an actor who was socially engaging (versus neutral) whilst demonstrating an action [112].

#### Autism-related similarities and differences in visual social attention

Though studies on expression discrimination (e.g. fragile X syndrome [79], 22q11.2 deletion syndrome [88]), social preference (e.g. Angelman syndrome [93], Phelan-McDermid syndrome [64]), gaze-following (e.g. Williams syndrome [109]) false-belief reasoning (i.e. Williams syndrome [114]) highlighted similarities between people with ID and autistic comparison groups comparable on chronological age and/or developmental, few studies considered how visual social attention may vary within ID groups by comparing those with co-occurring autism (non-syndromic ID [73, 101], Phelan-McDermid syndrome [94]). In addition, studies rarely analysed how visual social attention may be associated with clinical variables, such as autism characteristics (e.g. in Phelan-McDermid syndrome [99]), despite frequent discussion of how social-cognitive differences may underly social behaviour in ID groups.

### Meta-analysis

An exploratory meta-analysis was conducted to see whether visual social attention during studies of social cognition in ID correlated with degree of autism characteristics presented on clinical assessment tools. As no previous meta-analyses have explored this relationship, and there were limited data available within the reviewed literature (*k* = 16), effect sizes from a variety of eye-tracking studies measuring different social-cognitive abilities were included. Across studies, the visual social attention variable captured allocation of gaze upon pre-defined areas of interest that were considered to be ‘socially salient’ regions (SSRs) of the stimuli (see Table 2). Larger scores indicate increased visual attention on SSRs. The dependent variable for autism characteristics was total score on either a standardised screening questionnaire (i.e. Social Responsiveness Scale [SRS; [75]], Social Communication Questionnaire [SCQ; [76]], Gilliam Autism Rating Scale [GARS; [77]) or direct observational assessment (ADOS; [74]). Higher scores on these measures suggest a greater frequency and/or severity of autism characteristics.

Data were included for studies on fragile X (FXS; *k* = 7 [63, 65, 67–69, 71, 72]; 43.75%), Cornelia de Lange (CdLS; *k* = 2 [66, 67], 12.5%), Rubinstein-Taybi (RTS; *k* = 2 [66, 67]; 12.5%), Williams (WS; *k* = 1 [70]; 6.25%), Phelan-McDermid (PMS; *k* = 1 [64]; 6.25%) and Angelman (AS; *k* = 1 [93]; 6.25%) syndromes, as well as non-syndromic ID (nsID; *k* = 2 [69, 73]; 12.5%). Three articles (63,79,103) included subgroups of people with ID of different aetiology (e.g. CdLS & FXS) within the same study; hence, to allow consideration of ID aetiology in the analysis, effect sizes for each group are included separately. Only effect sizes from the ID groups were analysed, as there was not sufficient data to perform the same analysis in comparison groups (e.g. autism, neurotypical) to compare effect sizes.

### Data analysis strategy

Data were analysed in R, using the Metafor package, version 3.6.2. A random effects model and quality effects model was used, due to the likelihood of uncontrolled factors including methodological heterogeneity across studies. The random effects model weights each study based on the number of participants and the variation from findings across the full set of studies. The DerSimonian and Laird [116] method of random effects modelling was used to calculate between studies variation (tau), as there was no indication that the distribution of effects was not normally distributed. An additional quality effects model [117] was also used to explore variation due to methodological factors; this model weighted studies according to their quality ratings (see Table 2), in addition to number of participants. It can be interpreted as the meta-analytic effect that would have been obtained had all the studies been of the same methodological quality as the highest quality in the review. Pearson’s *r* values were transformed to Fisher’s *Z* scores for analysis and converted back to *r* for interpretation.

## Methodological variation

Estimates of heterogeneity which can result from methodological variation in the studies were calculated using the Q statistic and *I*^2^ statistic. The degree of heterogeneity was classified as ‘low’ (25%), ‘medium’ (50%) and ‘large’ (75%) [118]. Given the diverse methodologies included, variation was expected in the reported effects to reflect the methodological differences between studies. Therefore, *I*^2^ < 75% was deemed acceptable for interpretation of a summary effect [119].

## Planned contrasts

**Fig. 2:**
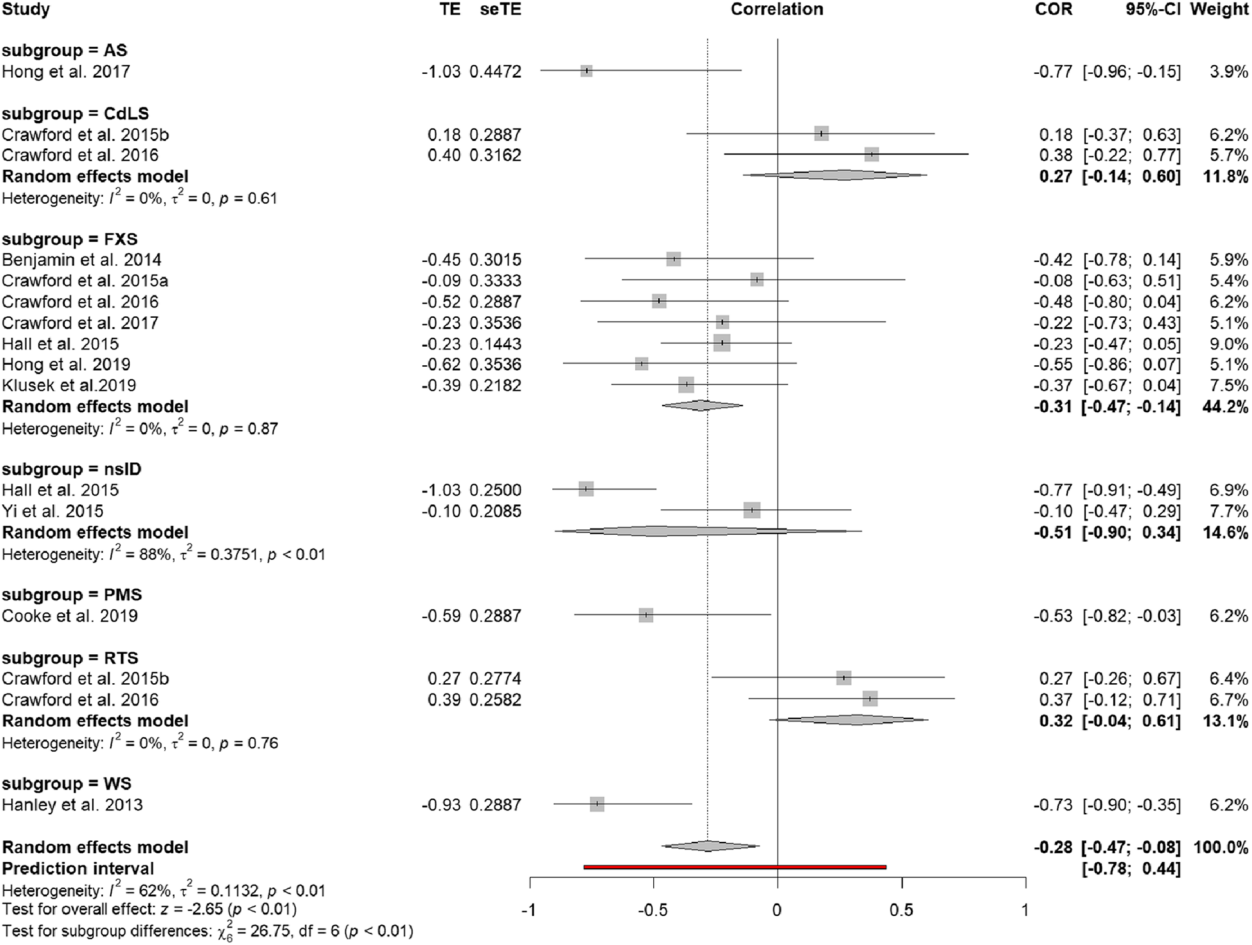
Forest plot of the relationship between visual attention on socially salient regions and autism characteristics

Subgroup analysis was applied from the outset to account for the different ID groups, to support ease of interpretation of the forest plot (see Fig. 2.). However, given that the number of effect sizes within each subgroup is ≤ four, there was not sufficient statistical power to conclude meaningful differences between each of the ID groups [120]. Instead, subgroup analyses were conducted on the following categorical moderator variables:

1. A group moderator variable was used to distinguish (a) FXS (*k* = 7) from (b) *other* ID groups (AS, CdLS, nsID, PMS, RTS, WS) (*k* = 9), given a high proportion of the effect sizes included were from people with FXS. Therefore, it was important to compare effect sizes from FXS to other ID groups, to assess these groups’ independent contributions to the overall effect.
2. A methodological moderator variable for measure of autism characteristics, categorised as (a) screening questionnaires (SCQ, SRS, GARS; *k* = 11) and (b) direct observational assessment (ADOS; *k* = 5) was used. Screening questionnaires are considered a less sensitive measure of autism characteristics than the ADOS (120) in ID. It was speculated this could result in a weaker effect.

Summary effects and associated heterogeneity measures were calculated for each of the subgroup analyses. It was not possible to control for other clinical variables such as IQ, adaptive functioning, social functioning, or other behavioural outcomes which frequently co-occur with autism (e.g. anxiety, ADHD) within the analyses, due to data availability and variability in methodology.

### Overall effect size

A total of 16 effect sizes were included, to inform a pooled effect size with data from a total of 283 participants. Results of the random effects model indicated that there was a negative correlation between visual attention on SSRs of the stimuli and autism characteristics, *r* = −.28, (95% confidence interval [CI −.47, −.08]), which was significantly different from zero (*z* = −2.65; *p* < .001). A significant level of heterogeneity (medium) was observed, (*Q* = 39.21, *df* = 15, *p* < .001, *I*^2^ = 61.7%). This was expected, given the various methodologies included, and was deemed reasonable as it fell below the cut-off of 75%. Results of the quality effects model returned a slightly smaller estimate of the correlation, *r* = −.25 (95% CI [−.47, −.03]), in which a significant level of heterogeneity (medium) was also observed (*Q* = 39.20, *df* = 15, *p* < .001, *I*^2^ = 61.7%). Visual inspection of the forest plot (see Fig. 2.) revealed preliminary evidence that in specific ID groups the direction of the effect was reversed, although confidence intervals spanned zero. For instance, in CdLS (*k* = 2) the pooled effect size was *r* = .27 (95% CI [−.14, .60]) and in RTS (*k* = 2) the pooled effect size was *r* = .32 (95% CI [−.04, .61]). Due to the small number of effect sizes available for these groups, the significance of these subgroup differences cannot be determined. Overall, estimates indicate a significant association between reduced visual attention on SSRs of the stimuli and higher autism characteristics across most ID groups.

### Subgroup analyses

There was no significant difference between the pooled effect size for FXS and other ID groups (*Q* = .11, *df* = 1, *p* = .756). However, in FXS there was a trend towards a greater negative correlation between visual attention on SSRs and autism characteristics (*r* = −.31 (95% CI [−.47, −.14], *k* = 7) with smaller heterogeneity (*I*^2^ = 0% [*p* = .878]), in comparison to other ID groups where the pooled effect was slightly smaller (*r* = −.25 [95% CI (−.57, .14), *k* = 9]) and there was much larger heterogeneity (*I*^2^ = 78% [*p* < .001]). There was no significant difference between the pooled effect size from studies which used screening questionnaires compared to direct observational assessment (*Q* = 1.16, *df* = 1, *p* = .282). However, there was a trend towards a smaller negative correlation between visual attention on SSRs and autism characteristics on screening questionnaires (*r* = −.23 (95% CI [−.49, .07], *k* = 11), with larger heterogeneity (*I*^2^ = 72% [*p* < .001]) than for direct observational assessment where the correlation was greater (*r* = −.42 (95% CI [−.60, −.19], *k* = 5) and heterogeneity was smaller (*I*^2^ = 0% [*p* = .941]). Notably, many of the studies in FXS used direct assessment to measure autism characteristics (*k* = 4), and in most of the other ID groups, screening questionnaires were used. Therefore, it is not currently possible to account for possible influences of these moderating factors by estimating their contribution individually.

### Publication bias

**Fig. 3:**
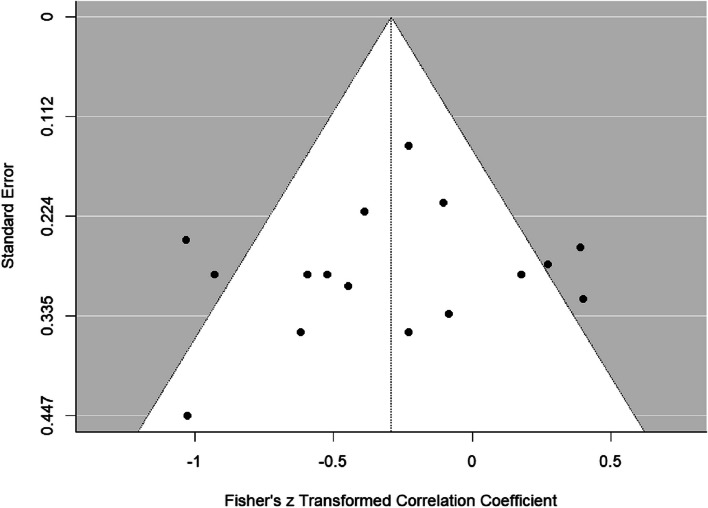
Funnel plot indicating the symmetry of the data in relation to publication bias

Publication bias was explored through inspection of funnel plots and the use of a trim and fill procedure which estimates the number of missing studies due to publication bias and calculates an adjusted effect size for the analysis. The funnel plot of the correlation between standard error by Fisher’s *Z* for the overall effect size is presented in Fig. 3. Visual inspection of the funnel plot demonstrated little evidence of publication bias, as the plot resembled a somewhat symmetrical (inverted) funnel with much of the study level effect within the boundaries. This conclusion was backed by Egger and colleagues’ [121] linear regression test of funnel plot asymmetry (bias = −.76, *t*(14) = −.50, *p* = .627). Using Duval and Tweedie’s [122] ‘Trim and Fill’ method no imputed studies were added. The uncorrected estimate of the effect size is −.29 (95% CI [ −.51, −.08). As there is little evidence of publication bias, the overall effect size value describing the relationship between visual attention on SSRs of the stimuli and autism characteristics can be seen to be reasonably robust.

## Discussion

To date, relatively little is known about social cognition in people with ID, particularly regarding whether these abilities are associated with autism characteristics. A limitation has been that traditional social-cognitive tasks place demands on domain-general cognition and language [26]. In autism research, eye-tracking technology has offered an effective method of evaluating social-cognitive abilities, independent of language ability (e.g.[7–9]), and indicated an association between visual social attention and autism characteristics (e.g. [41]). Here, we provided an account of research which has used eye-tracking paradigms to study social cognition in people with ID. An exploratory meta-analysis was used to estimate the degree to which visual attention to SSRs of the stimuli during these tasks may be related to degree of autism characteristics presented on clinical assessment tools.

### Summary of findings

Eye-tracking technology was used to measure different social-cognitive abilities across syndromic and non-syndromic ID groups. A range of infants, children and adults were studied. Samples were predominantly characterised by individuals with a mild to moderate degree of ID, although the range of IQ and adaptive behaviour scores reported across the studies indicate that samples were inclusive of individuals across a range of ability levels. There was also an example here of successful inclusion of those with severe to profound ID [91]. These findings speak to the way in which eye-tracking technology can support inclusion of people with ID of different ages and ability in social-cognitive research. Although there is preliminary evidence (*N* = 8) to suggest that those with high levels of hyperactivity and greater social motivation difficulties (as defined by higher scores on the SRS) may find it challenging to sustain their attention throughout the task [91]. Methods of supporting engagement should be considered during experimental design, as an attempt to minimise exclusion and improve sample validity. Examples include using minimal (e.g. [2–5]) calibration points, short (< five minutes) task length, attention grabbers, and mobile eye-trackers tolerant to head movements. Notably, many studies required participants to provide explicit responses (e.g. verbally identify emotional expressions) alongside completion of the eye-tracking task [70, 78–90]. Such demands are likely to limit who can participate—particularly those with severe to profound ID. Therefore, passive-viewing paradigms (e.g. [65, 79]), used alongside tasks with minimal (if any) explicit demands, may improve accessibility. Co-occurrence of visual impairment and/or physical disability (e.g. scoliosis) can also limit participation, as is the case for eye-tracking research more broadly [123], and therefore should be expected.

Studies highlighted differences in spontaneous expression discrimination and facial recognition across ID groups. This may be partly due to shorter scan paths and longer fixations, also described as ‘sticky fixations’ [114], resulting in limited exploration of stimuli. Studies which explored the specificity of these gaze patterns, comparing responses on social versus non-social tasks, highlighted that a general visual processing difference may underly atypical visual social attention [87, 88, 100]. Regardless, many studies indicated that atypical attentional capture and appraisal of social information impacted response to social cues (e.g. gaze-following) and people’s ability to make explicit inferences about mental states [70, 73, 90, 98, 101]—demonstrating the significance of visual social attention for social-cognitive processes.

Furthermore, the gaze patterns seen on social-cognitive tasks were reminiscent of social behaviours described in specific syndromes. For instance, people with fragile X (a syndrome characterised by social avoidance; 32), tended to fixate less on the eye region of faces and were initially hesitant to look towards people. Likewise, in syndromes associated with hypersociability, such as Down syndrome and Williams syndrome [29, 32], a preference for faces and increased social content was described. Thus, differences in gaze patterns appear to parallel notable features of specific behavioural phenotypes.

Few studies considered how visual attention may vary within ID groups by comparing those with co-occurring autism or analysed the association between visual social attention and clinical variables, such as autism characteristics, despite frequent discussion of how social-cognitive differences may underly social behaviour in ID groups. The meta-analysis provided preliminary evidence of a relationship between reduced visual attention to SSRs of the stimuli and a greater degree of autism characteristics across people with ID. The range of effect sizes were similar in direction and size as the relationship between visual social attention and autism characteristics evident in previous research studying autistic people (e.g. [39, 41]). It is possible that the relationship shown here may be moderated by factors such as the aetiology of ID and/or the type of clinical assessment tool used. Though subgroup analyses highlighted some potential indications of this, the small number of effects and the highly confounded nature of these variables across studies prevent a firm drawing of conclusions.

More research within syndromic and non-syndromic ID is needed, to establish whether the strength and direction of the relationship seen here varies across ID groups. Current evidence, whilst limited, raises the intriguing possibility that in some groups—Cornelia de Lange and Rubinstein-Taybi syndrome—increased visual attention to SSRs of the stimuli may be related to *greater* autism characteristics. This should be investigated further and considered within the context of the heterogeneous autism profiles and divergent behavioural phenotypes (e.g. hypervigilance versus avoidance [67]) presented in these groups.

### Methodological heterogeneity, small sample sizes and data quality

The social-cognitive domain studied most often using eye-tracking was expression discrimination. However, synthesis of the methodology highlighted variability in eye-tracking protocols and heterogeneity of stimuli used. There was also very little research on other abilities, such as false-belief reasoning (*N* = 1), which has been researched extensively in regard to the neurotypical development of social cognition [124] and theorised to be a core difficulty associated with autism [53, 125]. Furthermore, small sample size is a limitation of many of the studies reviewed, resulting in relatively low power and reduced replicability. Small sample sizes are also likely to be impacted by individual differences (e.g. age, co-occurring diagnoses) which are often broader in ID than that observed in neurotypical samples [126, 127]. Together, this emphasises the importance of sharing eye-tracking stimuli and protocols, to reduce methodological heterogeneity, enable further analyses of pooled effect sizes, and encourage replication. Given that there has been a much larger focus on using eye-tracking technology to measure social cognition in autism research, collaboration between autism and ID researchers is key to developing a bank of open access, validated paradigms. In doing this, researchers should establish normative data, which would support efforts to explore the developmental trajectory of mechanisms underlying social cognition in ID.

It should also be noted that none of the studies provided metrics to describe the quality of eye movement data beyond calibration, such as accuracy values (i.e. the difference between the true gaze position and the gaze position recorded) and the proportion of data loss, indicating a need to improve adherence to minimal reporting standards (e.g. [128]). Researchers should work towards incorporating these metrics where possible, considering associations with participant characteristics (e.g. hyperactivity), to support efforts to understand the feasibility of eye-tracking in ID more broadly [123].

### Understanding the role of intellectual disability

The majority of the reviewed literature was on genetic syndromes, with Williams syndrome and fragile X syndrome being the groups studied most often. Surprisingly, there were relatively few studies in which a non-syndromic ID group were included, particularly those where a diagnosis of autism was reported. This may be, in part, due to ambiguity in the terminology used to describe autism co-occurring with ID. Some studies referred to samples as ‘low-functioning’, ‘minimally verbal’ or ‘severely’ autistic, in place of ID-specific descriptors—which, without evidence of co-occurring ID (e.g. IQ), led to exclusion from the review. With that being said, there is clearly a gap in current knowledge on social-cognitive processes in non-syndromic ID relative to syndromic ID, which should be explored further. A better understanding of what visual social attention is like in this group could support efforts to distinguish possible ID-, syndromic- and autism-specific social-cognitive profiles.

The degree to which associated ID may account for the relationship between visual attention on SSRs of the stimuli and autism characteristics is unclear. Limited data on IQ and/or adaptive functioning meant that degree of ID severity could not be explored as a factor within the meta-analysis. Although it should be noted that in studies where effect sizes were available for different ID groups [66, 67, 69], participants had been matched on adaptive functioning (ABC), yet there are clear differences in effect size and/or direction. For example, Crawford and colleagues [67] report a positive correlation between visual attention on SSRs and autism characteristics in Cornelia de Lange syndrome (ABC = 47.9 [SD =16.0]) and Rubinstein-Taybi syndrome (ABC = 47.8 [SD = 14.6]), whereas in fragile X syndrome (ABC = 51.3 [SD = 17.4]) this correlation was negative. The opposite association presented in these genetic syndromes indicates that the relationship between visual attention on SSRs and autism characteristics cannot be entirely attributed to adaptive functioning. Further research is needed to establish the extent to which ID severity, alongside other associated characteristics (e.g. ADHD, anxiety), contributes to the relationship between visual social attention and autism characteristics. It is particularly important to understand whether the nature of this association varies between genetic syndromes, given ongoing efforts to disentangle the heterogeneity of autism from characteristics inherent to the broader behavioural phenotype presented [129].

### Visual social attention and the dyad of autism characteristics

The strength of association between visual social attention and autism characteristics in ID may differ in relation to social communication versus restricted and repetitive behaviour sub-scores on autism assessment tools. Studies with autistic children have reported a significant negative correlation between visual social attention and scores on the social affect subdomain of the ADOS (e.g. [130]). Yet, there is no association for the restricted and repetitive behaviour subdomain [131–133], whereas non-social visual attention in autism has been found to be strongly associated with restricted and repetitive behaviours [134]. These findings illustrate the ‘fractionation’ of autism characteristics at the cognitive level [135]. Here, we used total scores from clinical assessments of autism, due to there being limited data available. As restricted and repetitive behaviours are included alongside social communication difficulties in the total score, it is possible that the reported effect is weaker than it may be for social communication alone. To gain insight into the specificity of visual social attention and how it may be indicative of differences at the behavioural level in ID, further work is needed to establish whether the association is greater for social communication difficulties specifically. It is also important to consider the extent to which the relationship with autism characteristics is subserved by differences in visual attention more generally. That is, whether a high level of restricted and repetitive behaviours relate to the more restricted scan paths and ‘sticky fixations’ reported in ID groups.

## Conclusions

Eye-tracking can be used as an accessible tool to measure more subtle social-cognitive processes among a range of people with ID. The reviewed literature highlighted differences in how people with ID attend to social stimuli compared to neurotypical comparison groups, and some similarities to autistic people. Interestingly, in genetic syndromes, some gaze patterns appear to parallel notable features of specific behavioural phenotypes. The meta-analysis provides preliminary evidence of a relationship between reduced visual social attention and a greater degree of autism characteristics on clinical assessment tools across ID groups. Together, these findings demonstrate that eye-tracking is sensitive to detecting discrete social-cognitive processes in people with ID, which appear associated with behavioural variability. Fine-grained measurement of social cognition could lead to improved understanding of autism and broader social differences presented by people with ID. Future research should seek to strengthen conclusions regarding visual social attention and the nature of association with autism characteristics, accounting for ID severity and other co-occurring conditions (e.g. ADHD, anxiety), in both syndromic and non-syndromic ID groups.

## Data Availability

The data that support the findings of this study are available from the corresponding author upon reasonable request.

## Footnotes

1 The term autism has been chosen over the diagnostic term autism spectrum disorder (ASD) wherever possible to reflect the view that autism is a difference rather than a dysfunction [17]. This is consistent with the neurodiversity perspective [18] and the deficit-as-difference conception of autism [19]. The identity-first phrasing ‘autistic people’ is also used, as it is reported to be the preferred term by the autism community [20].

2 In most cases, autism is diagnosed in people who do not have a known genetic syndrome. Similarly, some people with intellectual disability do not have a known genetic syndrome. In this paper we have used the term ‘non-syndromic’ to reflect such cases.

3 Groups with scores on IQ, adaptive functioning, verbal and/or non-verbal abilities which were not statistically different from the ID groups are described here as having a similar developmental level. The specific measures used to compare and/or match groups are reported in Table 2.

## References

[1] Bauminger N, Schorr Edelsztein H, Morash J. Social Information Processing and Emotional Understanding in Children with LD. 2005; J Learn Disabil. 2005; 10.1177/00222194050380010401 15727328

[2] Adolphs R, Minzenberg M (2009). Social Cognition. Handb Neurosci Behav Sci..

[3] Allison T, Puce A, McCarthy G (2000). Social perception from visual cues: role of the STS region. Trends Cogn Sci..

[4] Schultz RT (2005). Developmental deficits in social perception in autism: the role of the amygdala and fusiform face area. Int J Dev Neurosci..

[5] Boraston Z, Blakemore SJ (2007). The application of eye-tracking technology in the study of autism. J Physiol..

[6] Frazier TW, Strauss M, Klingemier EW, Zetzer EE, Hardan AY, Eng C (2017). A Meta-Analysis of Gaze Differences to Social and Nonsocial Information Between Individuals With and Without Autism. J American Academy of Child and Adol Psych..

[7] Guillon Q, Hadjikhani N, Baduel S, Rogé B (2014). Visual social attention in autism spectrum disorder: Insights from eye tracking studies. Neuro and Biobehav Rev..

[8] Mastergeorge AM, Kahathuduwa C, Blume J (2020). Eye-Tracking in Infants and Young Children at Risk for Autism Spectrum Disorder: A Systematic Review of Visual Stimuli in Experimental Paradigms. J Autism Dev Disord..

[9] Chita-Tegmark M (2016). Social attention in ASD: A review and meta-analysis of eye-tracking studies. Res Dev Disabil..

[10] Callenmark B, Kjellin L, Rönnqvist L, Bölte S (2014). Explicit versus implicit social cognition testing in autism spectrum disorder. Autism..

[11] Pelphrey K, Adolphs R, Morris JP (2004). Neuroanatomical substrates of social cognition dysfunction in autism. Ment Retard Dev Disabil Res Rev.

[12] Sasson NJ, Nowlin RB, Pinkham AE, Sasson N (2012). Social cognition, social skill, and the broad autism phenotype. Autism..

[13] Bishop-Fitzpatrick L, Mazefsky CA, Eack SM, Minshew NJ (2017). Correlates of social functioning in autism spectrum disorder: The role of social cognition. Res Autism Spectr Disord..

[14] Sasson NJ, Morrison KE, Kelsven S, Pinkham AE (2020). Social cognition as a predictor of functional and social skills in autistic adults without intellectual disability. Autism Res..

[15] Russell G, Mandy W, Elliott D, White R, Pittwood T, Ford T (2019). Selection bias on intellectual ability in autism research: A cross-sectional review and meta-analysis. Mol Autism..

[16] American Psychological Association (APA). DSM-5. Diagnostic Statistical Manual 5. 2013.

[17] Bottema-Beutel K, Kapp SK, Lester JN, Sasson NJ, Hand BN (2021). Avoiding Ableist Language: Suggestions for Autism Researchers. Autism Adulthood.

[18] Mcgee M (2012). Neurodiversity. Contexts..

[19] Kapp SK, Gillespie-Lynch K, Sherman LE, Hutman T (2013). Deficit, difference, or both? Autism and neurodiversity. Dev Psychol.

[20] Kenny L, Hattersley C, Molins B, Buckley C, Povey C, Pellicano E (2015). Which terms should be used to describe autism? Perspectives from the UK autism community. Autism..

[21] Charman T, Pickles A, Simonoff E, Chandler S, Loucas T, Baird G (2011). IQ in children with autism spectrum disorders: Data from the Special Needs and Autism Project (SNAP). Psychol Med..

[22] Richards C, Jones C, Groves L, Moss J, Oliver C (2015). Prevalence of autism spectrum disorder phenomenology in genetic disorders: A systematic review and meta-analysis. The Lancet Psychiatry..

[23] Zafeiriou DI, Ververi A, Dafoulis V, Kalyva E, Vargiami E (2013). Autism spectrum disorders: The quest for genetic syndromes. Am J Med Genet B Neuropsychiatr Genet.

[24] Ziats CA, Patterson WG, Friez M (2021). Syndromic Autism Revisited: Review of the Literature and Lessons Learned. Pediatr Neurol..

[25] Morrison KE, Pinkham AE, Kelsven S, Ludwig K, Penn DL, Sasson NJ (2019). Psychometric Evaluation of Social Cognitive Measures for Adults with Autism. Autism Res..

[26] Morel A, Peyroux E, Leleu A, Favre E, Franck N, Demily C (2018). Overview of social cognitive dysfunctions in rare developmental syndromes with psychiatric phenotype. Front Pediatr..

[27] Amadó A, Serrat E, Vallès-Majoral E (2016). The role of executive functions in social cognition among children with down syndrome: Relationship patterns. Front Psychol..

[28] Grant CM, Apperly I, Oliver C (2007). Is theory of mind understanding impaired in males with fragile X syndrome?. J Abnorm Child Psychol..

[29] Tager-Flusberg H, Skwerer DP, Joseph RM (2006). Model syndromes for investigating social cognitive and affective neuroscience: a comparison of autism and Williams syndrome. Soc Cogn Affect Neurosci..

[30] van der Fluit F, Gaffrey MS, Klein-Tasman BP (2012). Social cognition in Williams syndrome: Relations between performance on the social attribution task and cognitive and behavioral characteristics. Front Psychol..

[31] Niego A, Benítez-Burraco A (2020). Autism and Williams syndrome: truly mirror conditions in the socio-cognitive domain?. Int J of Dev Dis..

[32] Awan N, Pearson E, Shelley L, Greenhill C, Tarver J, Waite J (2022). The behavioral phenotype of Rubinstein-Taybi syndrome: A scoping review of the literature. Am J Med Genet Part A..

[33] Wilde L, Mitchell A, Oliver C (2016). Differences in Social Motivation in Children with Smith-Magenis Syndrome and Down Syndrome. J Autism Dev Disord..

[34] Crawford H, Moss J, Groves L, Dowlen R, Nelson L, Reid D (2020). A Behavioural Assessment of Social Anxiety and Social Motivation in Fragile X, Cornelia de Lange and Rubinstein-Taybi Syndromes. J Autism Dev Disord..

[35] Bozhilova N, Welham A, Adams D, Bissell S, Bruining H, Crawford H (2023). Profiles of autism characteristics in thirteen genetic syndromes: a machine learning approach. Mol Autism..

[36] Moss J, Howlin P (2009). Autism spectrum disorders in genetic syndromes: Implications for diagnosis, intervention and understanding the wider autism spectrum disorder population. J of Int Dis Res..

[37] Warner G, Moss J, Smith P, Howlin P (2014). Autism Characteristics and Behavioural Disturbances in ∼ 500 Children with Down’s Syndrome in England and Wales. Autism Res..

[38] Ellis K, Lewington P, Powis L, Oliver C, Waite J, Heald M (2020). Scaling of Early Social Cognitive Skills in Typically Developing Infants and Children with Autism Spectrum Disorder. J Autism Dev Disord..

[39] Chawarska K, Macari S, Shic F (2013). Decreased spontaneous attention to social scenes in 6-month-old infants later diagnosed with autism spectrum disorders. Biol Psychiatry..

[40] Jones W, Klin A (2013). Attention to eyes is present but in decline in 2–6-month-old infants later diagnosed with autism. Nature..

[41] Bacon EC, Moore A, Lee Q, Carter Barnes C, Courchesne E, Pierce K (2020). Identifying prognostic markers in autism spectrum disorder using eye tracking. Autism..

[42] de Wit TCJ, Falck-Ytter T, von Hofsten C (2008). Young children with Autism Spectrum Disorder look differently at positive versus negative emotional faces. Res Autism Spectr Disord..

[43] Del Valle Rubido M, Hollander E, McCracken JT, Shic F, Noeldeke J, Boak L (2020). Exploring Social Biomarkers in High-Functioning Adults with Autism and Asperger’s Versus Healthy Controls: A Cross-Sectional Analysis. J Autism Dev Disord..

[44] Nayar K, Shic F, Winston M, Losh M (2022). A constellation of eye-tracking measures reveals social attention differences in ASD and the broad autism phenotype. Mol Autism..

[45] Goold S, Murphy MJ, Goodale MA, Crewther SG, Laycock R (2023). Faster social attention disengagement in individuals with higher autism traits..

[46] Dijkhuis R, Gurbuz E, Ziermans T, Staal W, Swaab H (2019). Social attention and emotional responsiveness in young adults with Autism. Front Psychiatry..

[47] Speer L, Cook A, McMahon W, Clark E (2007). Face processing in children with autism: Effects of stimulus contents and type. Autism..

[48] Bradshaw J, Shic F, Holden AN, Horowitz EJ, Barrett AC, German TC (2019). The Use of Eye Tracking as a Biomarker of Treatment Outcome in a Pilot Randomized Clinical Trial for Young Children with Autism. Autism Res..

[49] Shic F, Naples AJ, Barney EC, Chang SA, Li B, Mcallister T (2022). The Autism Biomarkers Consortium for Clinical Trials: evaluation of a battery of candidate eye-tracking biomarkers for use in autism clinical trials. Mol Autism..

[50] Senju A (2012). Spontaneous theory of mind and its absence in autism spectrum disorders. Neuroscientist..

[51] Wimmer H, Perner J (1983). Beliefs about beliefs: Representation and constraining function of wrong beliefs in young children’s understanding of deception. Cognition..

[52] Schuwerk T, Vuori M, Sodian B (2015). Implicit and explicit Theory of Mind reasoning in autism spectrum disorders: The impact of experience. Autism..

[53] Senju A, Southgate V, White S, Frith U (2009). Mindblind eyes: An absence of spontaneous theory of mind in Asperger syndrome. Science..

[54] Tager-Flusberg H, Plesa Skwerer D, Joseph RM, Brukilacchio B, Decker J, Eggleston B/ et al Conducting research with minimally verbal participants with autism spectrum disorder Autism. 2016; 101177/1362361316654605 10.1177/1362361316654605 PMC6988898 27354431

[55] Tager-Flusberg H, Kasari C (2013). Minimally Verbal School-Aged Children with Autism Spectrum Disorder: The Neglected End of the Spectrum. Autism Res..

[56] Page MJ, McKenzie JE, Bossuyt PM, Boutron I , Hoffmann TC, Mulrow CD (2021). The PRISMA 2020 statement: an updated guideline for reporting systematic reviews. Int J Surg.

[57] McGowan  J, Sampson  M, Salzwedel  DM, Cogo  E, Foerster  V, Lefebvre  C (2016). PRESS Peer Review of Electronic Search Strategies: 2015 Guideline Statement. J Clin Epidemiol.

[58] Riby DM, Hanley M, Kirk H, Clark F, Little K, Fleck R (2014). The interplay between anxiety and social functioning in Williams syndrome. J Autism Dev Disord..

[59] Cross EM, Hare DJ (2013). Behavioural phenotypes of the mucopolysaccharide disorders: a systematic literature review of cognitive, motor, social, linguistic and behavioural presentation in the MPS disorders. J Inherit Metab Dis..

[60] Masson R, Brusa C, Scoto M, Baranello G (2021). Brain, cognition, and language development in spinal muscular atrophy type 1: a scoping review. Dev Med Child Neurol..

[61] Pearson E, Wilde L, Heald M, Royston R, Oliver C (2019). Communication in Angelman syndrome: a scoping review. Dev Med Child Neurol..

[62] Hirai M, Muramatsu Y, Mizuno S, Kurahashi N, Kurahashi H, Nakamura M (2017). Preserved search asymmetry in the detection of fearful faces among neutral faces in individuals with Williams syndrome revealed by measurement of both manual responses and eye tracking. J Neurodev Disord..

[63] Benjamin DP, Mastergeorge AM, McDuffie AS, Kover ST, Hagerman RJ, Abbeduto L (2014). Effects of labeling and pointing on object gaze in boys with fragile X syndrome: An eye-tracking study. Res Dev Disabil..

[64] Cooke J, San José Cáceres A, Mason, L, Faulkner J, Crawley D, Hayward H, Ahmad J, Oakley B, Ellis C, Murphy D, Loth E. The Neurocognitive Profile of Individuals with Phelan-McDermid Syndrome (PMS): Individual Differences and Autism Spectrum Disorder (ASD). INSAR. 2013;

[65] Crawford H, Moss J, Anderson GM, Oliver C, McCleery JP (2015). Implicit Discrimination of Basic Facial Expressions of Positive/Negative Emotion in Fragile X Syndrome and Autism Spectrum Disorder. Am J Intellect Dev Disabil..

[66] Crawford H, Moss J, McCleery JP, Anderson GM, Oliver C (2015). Face scanning and spontaneous emotion preference in Cornelia de Lange syndrome and Rubinstein-Taybi syndrome. J Neurodev Disord..

[67] Crawford H, Moss J, Oliver C, Elliott N, Anderson GM, McCleery JP (2016). Visual preference for social stimuli in individuals with autism or neurodevelopmental disorders: An eye-tracking study. Mol Autism..

[68] Crawford H, Moss J, Oliver C, Riby D (2017). Differential effects of anxiety and autism on social scene scanning in males with fragile X syndrome. J Neurodev Disord..

[69] Hall SS, Frank MC, Pusiol GT, Farzin F, Lightbody AA, Reiss AL (2015). Quantifying naturalistic social gaze in fragile X syndrome using a novel eye tracking paradigm. Am J Med Genet Part B Neuropsychiatr Genet..

[70] Hanley M, Riby DM, Caswell S, Rooney S, Back E (2013). Looking and thinking: How individuals with Williams syndrome make judgements about mental states. Res Dev Disabil..

[71] Hong MP, Eckert EM, Pedapati EV, Shaffer RC, Dominick KC, Wink LK (2019). Differentiating social preference and social anxiety phenotypes in fragile X syndrome using an eye gaze analysis: A pilot study. J Neurodev Disord..

[72] Klusek J, Moser C, Schmidt J, Abbeduto L, Roberts JE. A novel eye-tracking paradigm for indexing social avoidance-related behavior in fragile X syndrome. Am J Med Genet Part B Neuropsychiatr Genet. 2020;183(1):5–16. Available from: https://www.embase.com/search/results?subaction=viewrecord&id=L2002485488&from=export 10.1002/ajmg.b.32757 PMC6898737 31418535

[73] Yi L, Quinn PC, Feng C, Li J, Ding H, Lee K (2015). Do individuals with autism spectrum disorder process own- and other-race faces differently?. Vision Res..

[74] Lord C, Rutter M, DiLavore PC, Risi S, Gotham K, Bishop SL. Autism diagnostic observation schedule, 2nd ed. (ADOS-2). 2012.

[75] Constantino JN, Gruber CP. The Social Responsivness Scale (2^nd^ Ed). Western Psychological Services; 2012.

[76] Rutter M, Bailey A, Lord C. The Social Communication Questionnaire: Manual. Los Angeles: Western Psychological Services; 2003.

[77] Gilliam JE. Gilliam Autism Rating Scale–Third Edition (GARS-3). Autisn, Pro-Ed. 2014.

[78] Campbell L, McCabe K, Leadbeater K, Schall U, Loughland C, Rich D (2010). Visual scanning of faces in 22q11.2 deletion syndrome: Attention to the mouth or the eyes?. Psychiatry Res.

[79] Dalton KM, Holsen L, Abbeduto L, Davidson RJ (2008). Brain function and gaze fixation during facial-emotion processing in fragile X and autism. Autism Res..

[80] Debladis J, Valette M, Strenilkov K, Mantoulan C, Thuilleaux D, Laurier V (2019). Face processing and exploration of social signals in Prader-Willi syndrome: A genetic signature. Orphanet J Rare Dis..

[81] Djukic A, Rose SA, Jankowski JJ, Feldman JF (2014). Rett syndrome: Recognition of facial expression and its relation to scanning patterns. Pediatr Neurol..

[82] Farzin F, Rivera SM, Hessl D (2009). Brief report: Visual processing of faces in individuals with fragile X syndrome: An eye tracking study. J Autism Dev Disord..

[83] Farzin F, Scaggs F, Hervey C, Berry-Kravis E, Hessl D (2011). Reliability of eye tracking and pupillometry measures in individuals with fragile X syndrome. J Autism Dev Disord..

[84] Franchini  M, Schaer  M, Glaser  B, Kott-Radecka  M, Debanné  M, Schneider  M (2016). Visual processing of emotional dynamic faces in 22q11.2 deletion syndrome. J Intellect Disabil Res.

[85] Gomez A, Costa M, Lio G, Sirigu A, Demily C (2020). Face first impression of trustworthiness in Williams Syndrome: Dissociating automatic vs decision based perception. Cortex..

[86] Kirk HE, Hocking DR, Riby DM, Cornish KM (2013). Linking social behaviour and anxiety to attention to emotional faces in Williams syndrome. Res Dev Disabil..

[87] McCabe  K, Rich  D, Loughland  CM, Schall  U, Campbell  LE (2011). Visual scanpath abnormalities in 22q11.2 deletion syndrome: Is this a face specific deficit?. Psychiatry Res.

[88] McCabe KL, Melville JL, Rich D, Strutt PA, Cooper G, Loughland CM (2013). Divergent patterns of social cognition performance in autism and 22q11.2 deletion syndrome (22q11DS). J Autism Dev Disord.

[89] Porter MA, Shaw TA, Marsh PJ (2010). An unusual attraction to the eyes in Williams-Beuren syndrome: A manipulation of facial affect while measuring face scanpaths. Cogn Neuropsychiatry..

[90] Shaw TA, Porter MA (2013). Emotion recognition and visual-scan paths in fragile X syndrome. J Autism Dev Disord..

[91] Hirai M, Muramatsu Y , Mizuno S, Kurahashi N, Kurahashi H , Nakamura M (2016). Intact attentional orienting towards inverted faces revealed by both manual responses and eye-movement measurement in individuals with Williams syndrome. J Intellect Disabil Res.

[92] Hirai M, Muramatsu Y, Mizuno S, Kurahashi N, Kurahashi H, Nakamura M (2016). Typical visual search performance and atypical gaze behaviors in response to faces in Williams syndrome. J Neurodev Disord..

[93] Hong MP, Guilfoyle JL, Mooney LN, Wink LK, Pedapati EV, Shaffer RC (2017). Eye gaze and pupillary response in Angelman syndrome. Res Dev Disabil..

[94] Ponson L, Gomot M, Blanc R, Barthelemy C, Roux S, Munnich A (2018). 22q13 deletion syndrome: communication disorder or autism? Evidence from a specific clinical and neurophysiological phenotype. Transl Psychiatry..

[95] Riby DM, Hancock PJB (2009). Do faces capture the attention of individuals with Williams syndrome or autism? Evidence from tracking eye movements. J Autism Dev Disord..

[96] Schwartzman JS, De Lima Velloso R, D’Antino MEF, Santos S (2015). The eye-tracking of social stimuli in patients with Rett syndrome and autism spectrum disorders: A pilot study. Arq Neuropsiquiatr..

[97] Vivanti G, Fanning PAJ, Hocking DR, Sievers S, Dissanayake C (2017). Social attention, joint attention and sustained attention in autism spectrum disorder and williams syndrome: Convergences and divergences. J Autism Dev Disord..

[98] Glaser  B, Debban  M, Ottet  M-C,  Vuilleumier  P,  Zesiger  P, Antonarakis  SE (2010). Eye gaze during face processing in children and adolescents with 22q11.2 deletion syndrome. Acad Child Adolesc Psychiatry.

[99] Guillory SB, Baskett VZ, Grosman HE, McLaughlin CS, Isenstein EL, Wilkinson E (2021). Social visual attentional engagement and memory in Phelan-McDermid syndrome and autism spectrum disorder: A pilot eye tracking study. J Neurodev Disord..

[100] Rose SA, Djukic A, Jankowski JJ, Feldman JF, Fishman I, Valicenti-Mcdermott M (2013). Rett syndrome: An eye-tracking study of attention and recognition memory. Dev Med Child Neurol..

[101] Yi L, Feng C, Quinn PC, Ding H, Li J, Liu Y (2014). Do individuals with and without autism spectrum disorder scan faces differently? A new multi-method look at an existing controversy. Autism Res..

[102] Zaharia A, Schneider M, Glaser B, Franchini M, Menghetti S, Schaer M (2018). Face processing in 22q112 deletion syndrome: Atypical development and visual scanning alterations. J Neurodev Disord..

[103] Guy J, Ng-Cordell E, Doherty BR, Duta M, Scerif G (2020). Understanding attention, memory and social biases in fragile X syndrome: Going below the surface with a multi-method approach. Res Dev Disabil..

[104] Liang J, Wilkinson K (2018). Gaze Toward Naturalistic Social Scenes by Individuals With Intellectual and Developmental Disabilities: Implications for Augmentative and Alternative Communication Designs. J Speech Lang Hear Res..

[105] Riby DM, Hancock PJB (2008). Viewing it differently: Social scene perception in Williams syndrome and Autism. Neuropsychologia..

[106] Riby D, Hancock PJB (2009). Looking at movies and cartoons: Eye-tracking evidence from Williams syndrome and autism. J Intellect Disabil Res..

[107] Wilkinson KM, Light J (2014). Preliminary study of gaze toward humans in photographs by individuals with autism, Down syndrome, or other intellectual disabilities: implications for design of visual scene displays. Augment Altern Commun..

[108] Williams TA, Porter MA, Langdon R (2013). Viewing social scenes: A visual scan-path study comparing fragile X syndrome and williams syndrome. J Autism Dev Disord..

[109] Riby DM, Hancock PJ, Jones N, Hanley M (2013). Spontaneous and cued gaze-following in autism and Williams syndrome. J Neurodev Disord..

[110] D’Souza D, D’Souza H, Johnson MH, Karmiloff-Smith A (2015). Concurrent relations between face scanning and language: A cross-syndrome infant study. PLoS One..

[111] Kleberg JL, Riby D, Fawcett C, Björlin Avdic H, Frick MA, Brocki KC, et al. Williams syndrome: reduced orienting to other’s eyes in a hypersocial phenotype. J Autism Dev Disord. 2022 Apr 20;1–12. Available from: https://link.springer.com/article/10.1007/s10803-022-05563-6 10.1007/s10803-022-05563-6 PMC9020553 35445369

[112] Vivanti G, Hocking DR, Fanning P, Dissanayake C. Social affiliation motives modulate spontaneous learning in Williams syndrome but not in autism. Mol Autism. 2016;7(1). Available from: https://www.embase.com/search/results?subaction=viewrecord&id=L612003273&from=export 10.1186/s13229-016-0101-0 PMC5015226 27610215

[113] Vivanti G, Hocking DR, Fanning P, Dissanayake C. The social nature of overimitation: Insights from Autism and Williams syndrome. Cognition [Internet]. 2017;161:10–8. Available from: https://www.embase.com/search/results?subaction=viewrecord&id=L614038993&from=export 10.1016/j.cognition.2017.01.008 28088702

[114] Van Herwegen J, Smith TJ, Dimitriou D (2015). Exploring different explanations for performance on a theory of mind task in Williams syndrome and autism using eye movements. Res Dev Disabil..

[115] D’Souza D, D’Souza H, Jones EJH, Karmiloff-Smith A (2020). Attentional abilities constrain language development: A cross-syndrome infant/toddler study. Dev Sci..

[116] DerSimonian R, Laird N (1986). Meta-analysis in clinical trials. Control Clin Trials..

[117] Doi S, Thalib L (2008). A Quality-Effects Model for Meta-Analysis. JSTOR..

[118] Higgins JPT, Thompson SG, Deeks JJ, Altman DG. Measuring inconsistency in meta-analyses. BMJ. 2003; 10.1136/bmj.327.7414.557 PMC192859 12958120

[119] Higgins JPT (2008). Commentary: Heterogeneity in meta-analysis should be expected and appropriately quantified. Int J Epidemiol..

[120] Cuijpers P, Griffin JW, Furukawa TA (2021). The lack of statistical power of subgroup analyses in meta-analyses: a cautionary note. Epidemiol Psychiatr Sci..

[121] Egger M, Smith GD, Schneider M, Minder C (1997). Bias in meta-analysis detected by a simple, graphical test. BMJ.

[122] Duval S, Tweedie R (2012). A Nonparametric, “Trim and Fill” Method of Accounting for Publication Bias in Meta-Analysis. J of American Stat Assoc..

[123] Csákvári J, Gyori M (2015). Applicability of standard eye-tracking technique in people with intellectual disability: methodological conclusions from a series of studies. Stud Health Technol Inform..

[124] Schneider D, Slaughter VP, Dux PE (2014). What do we know about implicit false-belief tracking?. Psychon Bull Rev..

[125] Gliga T, Senju A, Pettinato M, Charman T, Johnson MH (2014). Spontaneous belief attribution in younger siblings of children on the autism spectrum. Dev Psychol..

[126] Mazza MG, Rossetti A, Crespi G, Clerici M (2020). Prevalence of co-occurring psychiatric disorders in adults and adolescents with intellectual disability: A systematic review and meta-analysis. J Appl Res Intellect Disabil..

[127] Munir KM (2016). The co-occurrence of mental disorders in children and adolescents with intellectual disability/intellectual developmental disorder. Curr Opin Psychiatry..

[128] Holmqvist K, Örbom SL, Hooge IT, Niehorster DC, Alexander RG, Andersson R (2023). Eye tracking: Empirical foundations for a minimal reporting guideline. Behavior Research Methods..

[129] Jenner L, Richards C, Howard R, Moss J. Heterogeneity of autism characteristics in genetic syndromes: Key considerations for assessment and support. Current Developmental Disorders Reports. 2023; 132–46. 10.1007/s40474-023-00276-6 PMC10169182 37193200

[130] Jones W, Carr K, Klin A (2008). Absence of Preferential Looking to the Eyes of Approaching Adults Predicts Level of Social Disability in 2-Year-Old Toddlers With Autism Spectrum Disorder. Arch Gen Psychiatry..

[131] Kou J, Le J, Fu M, Lan C, Chen Z, Li Q (2019). Comparison of three different eye-tracking tasks for distinguishing autistic from typically developing children and autistic symptom severity. Autism Res..

[132] Nyström P, Thorup E, Bölte S, Falck-Ytter T (2019). Joint Attention in Infancy and the Emergence of Autism. Biol Psychiatry..

[133] Sasson NJ, Turner-Brown LM, Holtzclaw TN, Lam KSL, Bodfish JW (2008). Children with autism demonstrate circumscribed attention during passive viewing of complex social and nonsocial picture arrays. Autism Res..

[134] Sasson NJ, Touchstone EW (2014). Visual attention to competing social and object images by preschool children with autism spectrum disorder. J Autism Dev Disord..

[135] Brunsdon V, Happé F (2014). Exploring the ’fractionation’of autism at the cognitive level. Autism..

